# RORα-activated mitophagy attenuating hypoxic-ischemic encephalopathy via suppression of microglial cGAS-STING axis

**DOI:** 10.3389/fimmu.2025.1592737

**Published:** 2025-07-29

**Authors:** Lei Song, Haiyan Shen, Fei Hong, Weiyan Zhang, Hongyi Lu

**Affiliations:** Department of Pediatrics, Nantong First People’s Hospital (Second Affiliated Hospital of Nantong University), Nantong, Jiangsu, China

**Keywords:** hypoxic-ischemic encephalopathy, RORα, mitophagy, scRNA-seq, neuroinflammation, cGAS-STING pathway, NLRP3 inflammasome

## Abstract

**Introduction:**

Hypoxic-ischemic encephalopathy (HIE) involves neuroinflammation driven by microglial activation, yet regulatory mechanisms remain poorly defined. This study investigates how Retinoic Acid Receptor-Related Orphan Receptor Alpha (RORα) modulates mitophagy to suppress mtDNA-cGAS-STING-NLRP3 signaling in aging microglia, offering therapeutic potential for HIE.

**Methods:**

A multi-omics approach combining single-cell RNA sequencing (scRNA-seq) of an HIE rat model, Weighted Gene Co-Expression Network Analysis (WGCNA), and LASSO regression identified RORα as a pivotal regulator. *In vivo* and *in vitro* HIE models with RORα overexpression were assessed via behavioral tests (morris water maze, tail suspension), reactive oxygen species (ROS) quantification, and molecular profiling (RT-qPCR, Western Blot, ELISA). Mitophagy inhibitor 3-MA was used to validate pathway dependence.

**Results:**

Multi-omics integration revealed RORα as a hub gene linked to inflammatory and metabolic pathways. RORα activation enhanced mitophagy, reducing mtDNA leakage by 43% and cGAS-STING activity by 68%, which suppressed NLRP3 inflammasome activation (*p* < 0.01). This correlated with improved cognitive/motor function in HIE rats (*p* < 0.05) and attenuated ROS/IL-1β levels. Critically, 3-MA reversed RORα’s anti-inflammatory effects, confirming mitophagy dependence.

**Conclusion:**

RORα alleviates HIE by resolving microglial neuroinflammation through mitophagic inhibition of mtDNA-cGAS-STING-NLRP3 signaling. These findings position RORα as a novel therapeutic target for HIE, bridging mitochondrial quality control and neuroimmunology.

## Graphical Abstract

RORα Regulates the mtDNA-cGAS-sting signaling pathway by activating mitophagy, thereby inhibiting NLRP3 inflammasome activation and alleviating HIE.

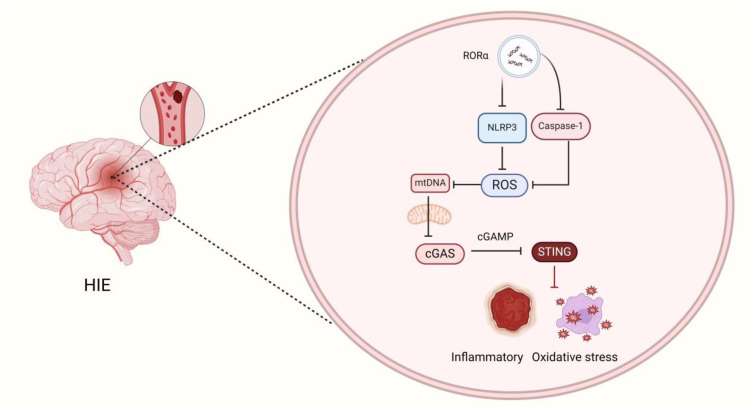

## Introduction

Hypoxic-ischemic encephalopathy (HIE) is a severe perinatal brain injury that significantly impairs long-term neurodevelopment in newborns. The pathogenesis of HIE is complex and involves multiple interwoven factors, with oxidative stress and inflammatory responses playing central roles in the disease process (1–4). During the perinatal period, newborns may experience hypoxic-ischemic events due to factors such as placental blood flow obstruction, prolonged labor, or asphyxia, leading to brain injury (5, 6). Clinically, HIE patients often present with cognitive and motor dysfunctions and may suffer from long-term sequelae such as epilepsy and intellectual disabilities, placing a significant burden on both the affected children and their families. Current treatments primarily include hypothermia therapy and supportive care, but these interventions are limited in effectiveness and cannot fully reverse brain damage (7, 8). Recent research into the pathophysiology of HIE suggests that targeting inflammatory and oxidative stress pathways may offer promising therapeutic strategies (9, 10). Thus, identifying new therapeutic targets in HIE holds considerable scientific and clinical importance (11, 12).

During the pathological progression of HIE, microglia, as the primary immune cells in the central nervous system, play dual roles in inflammatory response and tissue repair. Specific inflammasomes, such as the NLRP3 inflammasome, are crucial inflammatory activators in microglia. They can be triggered by endogenous molecules like mitochondrial DNA (mtDNA), initiating inflammatory cascades (13–16). Additionally, the cyclic GMP-AMP synthase (cGAS)-stimulator of interferon genes (STING) signaling pathway has a significant pathological role in brain injury. The enzyme cGAS detects mtDNA in the cytoplasm, activating the STING pathway, which initiates an inflammatory response and exacerbates neural damage (17). Numerous studies have demonstrated that the cGAS-STING pathway is activated in hypoxic-ischemic conditions like HIE, promoting the spread of inflammation (13, 18, 19). Therefore, strategies to effectively inhibit mtDNA release and the subsequent activation of the cGAS-STING pathway may offer new approaches to mitigate inflammatory damage in HIE (20).

Mitophagy is a critical mechanism for maintaining cellular homeostasis by clearing damaged mitochondria and has been shown to play a significant role in controlling inflammatory responses. Damaged mitochondria produce excessive reactive oxygen species (ROS) and release mtDNA, which can activate inflammasomes and the cGAS-STING signaling pathway, leading to the spread of inflammation (21, 22). Activation of mitophagy effectively removes these damaged mitochondria, thereby reducing the release of inflammatory factors (23, 24). However, research on how to activate mitophagy in HIE is limited, and the specific molecular regulatory mechanisms remain unclear (25, 26). Identifying molecules capable of activating mitophagy and modulating microglial inflammatory responses is crucial for suppressing inflammation in HIE pathology. Activation of mitophagy may offer a new strategy to reduce HIE-related inflammation and mitigate brain injury caused by hypoxia-ischemia at the molecular level (27).

Retinoic Acid Receptor-Related Orphan Receptor Alpha (RORα) is a nuclear receptor transcription factor recently identified for its potential to regulate immune and metabolic pathways. Studies indicate that RORα exerts anti-inflammatory and antioxidant effects by promoting mitophagy and reducing ROS production (28, 29). The expression of RORα in microglia is closely associated with immune functions, and its upregulation may suppress the excessive activation of the NLRP3 inflammasome, thereby alleviating inflammatory responses (30, 31). However, the precise mechanisms of RORα in HIE pathology remain unclear, particularly regarding whether it reduces inflammation cascades and oxidative stress in microglia by activating mitophagy and inhibiting the mtDNA-cGAS-STING pathway. Given RORα’s multifaceted roles in regulating inflammation and mitophagy, exploring its protective mechanisms in HIE holds substantial research value.

Based on the background described above, we proposed the following hypothesis: RORα may activate mitophagy to reduce mtDNA release and suppress cGAS-STING signaling activation, thereby inhibiting the excessive activation of the NLRP3 inflammasome in microglia. This ultimately attenuates oxidative stress and inflammatory responses associated with HIE. To validate this hypothesis, we first established a rat model of HIE and performed transcriptomic sequencing combined with Weighted Gene Co-expression Network Analysis (WGCNA) to identify key targets and verify the regulatory function of RORα in inflammation. Subsequently, we conducted *in vivo* experiments to evaluate the effects of RORα on microglial mitophagy, ROS production, and cytokine secretion. The impact of a mitophagy inhibitor on RORα’s anti-inflammatory efficacy was also assessed. Overall, this study aims to systematically elucidate the role and molecular mechanisms of RORα in HIE through multi-omics and experimental approaches, furthering the understanding of inflammation-induced injury in HIE. By validating the therapeutic relevance of RORα, this work provides theoretical and experimental support for future clinical intervention strategies for HIE.

## Materials and methods

### Experimental animals and grouping

Healthy 7-day-old Sprague Dawley (SD) rats (SPF-grade, equal numbers of males and females) were obtained from Beijing Vital River Laboratory Animal Technology Co., Ltd., China, and housed under standard conditions (12-hour light/dark cycle, with ad libitum access to food and water). All animal experiments in this study were conducted in accordance with the guidelines and regulations of our institution’s Animal Ethics Committee and received the necessary approvals. Efforts were made to minimize animal suffering and discomfort, as well as to reduce the number of animals used to the extent possible.

The rats were randomly assigned to four groups: Sham, HIE, HIE + RORα overexpression, and HIE + RORα + 3-MA. The sham group underwent surgical exposure of the right common carotid artery without ligation or hypoxic exposure. The HIE group underwent permanent ligation of the right common carotid artery followed by 90 minutes of exposure to an 8% O_2_ hypoxic environment. The HIE + RORα group received an intracerebroventricular injection of AAV-RORα at 1 hour post-HIE modeling. The HIE + RORα + 3-MA group received an intraperitoneal injection of 3-MA (20 mg/kg) 1 hour before HIE modeling, followed by the same AAV-RORα injection as above. For transcriptomic sequencing, 8 rats each from the sham and HIE groups were used. For single-cell RNA sequencing (scRNA-seq), 3 rats each from the HIE and HIE + RORα groups were used. For functional *in vivo* assessments, 5 rats were included in each group (**Supplementary Figure S1**).

### Neonatal HIE rat model

On postnatal day 7, neonatal rats were anesthetized with 2% isoflurane. After a midline neck incision, the right common carotid artery was exposed, double-ligated with 5.0 surgical sutures, and transected between the ligatures. The skin was sutured, and lidocaine was applied to the wound for local analgesia. The entire surgical procedure was completed within 5 minutes. Pups were returned to their dams for 1 hour, followed by hypoxic exposure in a chamber with 8% O_2_ and 92% N_2_ at 37°C for 2 hours. After hypoxia, the pups were returned to their dams. Rats showing abnormal motor function or >20% body weight loss within 24 hours post-surgery were excluded. Due to the lack of vital sign monitoring systems, the mortality rate was approximately 4%. Sham rats underwent arterial exposure only, without ligation or hypoxia (32, 33).

### Adeno-associated virus gene delivery

The AAV vectors were custom-produced by GeneChem (China), containing the pAAV-RORα construct driven by a CMV promoter, with a viral titer of 1×10¹³ vg/mL. A volume of 5 μL was injected into the lateral ventricle using a microsyringe at 1 hour post-HIE induction. The needle was retained for 10 minutes to prevent reflux, then slowly withdrawn, followed by standard wound disinfection and suturing (34).

### RNA extraction and sequencing

Brain tissues from both the control and HIE rat groups (eight samples per group) were collected, and total RNA was extracted using Trizol reagent (15596026, Invitrogen, Carlsbad, CA, USA). RNA concentration and purity were assessed with a Nanodrop2000 spectrophotometer (1011U, Nanodrop, USA). Only total RNA samples meeting the following quality criteria were used for further experiments: RNA Integrity Number (RIN) ≥ 7.0 and 28S:18S ratio ≥ 1.5.

Sequencing libraries were generated and sequenced by CapitalBio Technology (Beijing, China). For each sample, 5 µg of RNA was processed. Briefly, ribosomal RNA (rRNA) was removed from total RNA using the Ribo-Zero™ Magnetic Kit (MRZE706, Epicentre Technologies, Madison, WI, USA). The NEBNext Ultra RNA Library Prep Kit (#E7775, NEB, USA) was used to construct libraries for Illumina sequencing. RNA was fragmented in NEBNext First Strand Synthesis Reaction Buffer (5×) to produce fragments of approximately 300 base pairs (bp) in length. First-strand cDNA was synthesized with reverse transcriptase and random primers, followed by second-strand cDNA synthesis in dUTP Mix (10×) Second Strand Synthesis Reaction Buffer. cDNA ends were repaired, poly(A) tails were added, and sequencing adapters were ligated. The second cDNA strand was digested with USER Enzyme (#M5508, NEB, USA) to create strand-specific libraries. The library DNA was amplified, purified, and enriched via PCR. Library quality was assessed using an Agilent 2100 system and quantified with a KAPA Library Quantification Kit (KK4844, KAPA Biosystems). Finally, paired-end sequencing was conducted on a NextSeqCN500 sequencer (Illumina). A workflow diagram is provided in **Supplementary Figure S2**.

### Quality control of sequencing data and reference genome alignment

The quality of paired-end reads from raw sequencing data was assessed using FastQC software (v0.11.8). Preprocessing of the raw data was performed with Cutadapt (v1.18) to remove Illumina sequencing adapters and poly(A) tails. Reads with over 5% N content were filtered out using a custom Perl script. Reads with at least 70% of bases having a quality score above 20 were extracted with the FASTX Toolkit (v0.0.13), and paired-end sequences were repaired using BBMap. Finally, high-quality filtered reads were aligned to the rat reference genome using HISAT2 (v0.7.12).

The obtained gene expression data were then normalized using the Limma package in R (v3.48.3), with log2 transformation and quantile normalization applied to mitigate batch effects and technical noise. Differential expression analysis was conducted with DESeq2 (v1.32.0) and EdgeR (v3.34.1) to identify mRNAs significantly upregulated or downregulated in HIE patients. The criteria for selection were set at a *p*-value < 0.05 and |log_2_FoldChange| > 1.

### WGCNA

We calculated the Median Absolute Deviation (MAD) of each gene’s expression profile and excluded the top 50% of genes with the lowest MAD values to focus on genes with greater variability. Next, we applied the “goodSamplesGenes” function from the R package “WGCNA” to remove outlier genes and samples. Using WGCNA, we then constructed a scale-free co-expression network, setting the minimum height for the gene dendrogram at 9 and the sensitivity at 8. Modules with a dissimilarity measure below 0.2 were merged, resulting in four co-expression modules. We assessed the correlation between each module and the experimental groups using Pearson correlation analysis (*p* < 0.05). Genes within modules significantly associated with HIE were identified as disease-related genes and selected for further analysis (**Supplementary Figure S2**).

### Least absolute shrinkage and selection operator regression

In our bioinformatics research, we applied the LASSO regression to identify key genes associated with disease. First, we set a random seed to ensure experiment reproducibility and loaded the glmnet package to handle the dataset with numerous variables. We used the glmnet function to perform LASSO regression, modeling the data as a binary classification problem. The sample names were parsed with regular expressions to extract class labels as the response variable. The model evaluation involved plotting the model object and using cv.glmnet to perform cross-validation, identifying the optimal lambda value. Finally, genes corresponding to non-zero coefficients at the optimal lambda were considered key genes related to the disease state and were outputted as a result.

### Preparation of single-cell suspension

Brain tissues from three rats in the HIE group and three in the HIE + RORα overexpression group were collected and placed in ice-cold PBS. A single-cell suspension was prepared using mechanical and enzymatic digestion. First, the tissue was minced using a 70μm cell strainer (Falcon, USA). It was then incubated in a digestion solution containing 1mg/mL collagenase I (Worthington, USA) and 0.1mg/mL DNase I (Sigma-Aldrich, USA) at 37°C for 30 minutes, with gentle agitation every 5 minutes. After incubation, the solution was filtered through a 40μm cell strainer (Falcon, USA), treated with red blood cell lysis buffer (BioLegend, USA) for 5 minutes, and washed with PBS. The cells were resuspended in PBS containing 0.04% BSA, counted, and adjusted to a concentration of 1000 cells/μL.

### scRNA-seq library preparation and sequencing

Single-cell suspensions and library construction were performed using the 10x Genomics Chromium platform (10x Genomics, USA). Single-cell suspensions were loaded onto the Chromium Controller, and libraries were prepared following the Chromium Single Cell 3’ Reagent Kits v3 protocol (10x Genomics, USA). The constructed libraries were then sequenced on the Illumina NovaSeq 6000 platform (Illumina, USA), aiming for a target read depth of 50,000 reads per cell per sample.

### TSNE clustering analysis and cell communication

To reduce the dimensionality of the scRNA-seq dataset, we performed principal component analysis (PCA) on the top 2000 highly variable genes based on variance. Using the ElbowPlot function from the Seurat package, we selected the top 20 principal components (PCs) for downstream analysis. Primary cell subpopulations were identified through the Seurat function FindClusters, with the resolution set to the default value (res = 0.5). We then applied the t-SNE algorithm for nonlinear dimensionality reduction of the scRNA-seq data. Marker genes for various cell subpopulations were identified using the Seurat package, and cell annotation was conducted based on known lineage-specific marker genes and the online database CellMarker. Finally, cell communication analysis was performed with the “cellcall” package in R (**Supplementary Figure S3**).

### Differential analysis

Differential Gene Expression (DGE) analysis of Microglia across treatment groups was conducted using the FindMarkers function in the “Seurat” package in R. Genes with │logFC│ > 0.5 and *p*-value < 0.05 were considered differentially expressed. Visualization of the differential analysis results was performed using the “EnhancedVolcano” package in R, generating corresponding volcano plots.

### Gene ontology and Kyoto encyclopedia of genes and genomes enrichment analysis

Using the R package “ClusterProfiler,” we conducted GO and KEGG enrichment analyses for candidate targets or disease-associated differential genes, with a significance threshold set at *p* < 0.05. The GO analysis included assessments of biological processes (BP), molecular functions (MF), and cellular components (CC), identifying the primary cellular functions and signaling pathways enriched in the highly expressed genes of the sepsis group and in disease-associated differential genes. Based on *p*-values, we used the “ClusterProfiler” package to perform KEGG enrichment analysis on candidate targets and generated bubble plots to visualize the KEGG enrichment results.

### Construction of protein-protein interaction networks

The STRING database (http://www.string-db.org/) integrates experimental data, text-mining results from PubMed abstracts, information from various other databases, and bioinformatics-based predictions to investigate PPI. Using the STRING database, we analyzed significant overexpressed genes for protein interactions, setting the confidence score threshold at 0.4. The number of connected nodes for each protein was then statistically analyzed and visualized using R software. A higher number of connected nodes indicates a higher degree of centrality, suggesting a more central role within the network.

### Long-term neurological assessments

At day 10 post-surgery, five rats were randomly selected from each group for the following behavioral tests to evaluate long-term neurological function:

Morris Water Maze Test: The circular pool (diameter 120 cm, depth 30 cm, water temperature 22 ± 1°C) was divided into four quadrants, with a hidden platform placed 1 cm below the water surface in the third quadrant. All rats underwent 5 days of spatial navigation training prior to testing. During testing, rats were released from different quadrants, and the escape latency (time to find the hidden platform) was recorded. On day 6, the platform was removed for the probe trial. Parameters including swimming trajectory, time spent in the target quadrant, swim speed, and platform crossings were analyzed using the SMART video-tracking system (33).

Tail Suspension Test: Each rat was suspended by attaching Scotch tape approximately 1 cm from the tail tip and affixing it to a rod 50 cm above the surface. A small plastic tube was placed over the tail to prevent climbing. Immobility time was manually recorded over a 6-minute period. Rats were considered immobile only when they hung passively and remained completely still (35).

### Immunohistochemistry analysis

To assess the expression of RORα in a HIE rat model, IHC staining was performed. Brain tissue samples from the rat model were collected immediately post-surgery. All tissue samples were fixed in 4% paraformaldehyde (Sigma-Aldrich, USA) and embedded in paraffin before sectioning. Antigen retrieval was carried out using citrate buffer (pH 6.0) (Beyotime, China), followed by natural cooling at room temperature. Next, endogenous peroxidase activity was blocked by treating the sections with 3% hydrogen peroxide (Beyotime, China) for 10 minutes. The sections were then blocked with 10% normal goat serum (Gibco, USA) for 30 minutes and incubated overnight with a specific anti-RORα primary antibody (1:200, Abcam, UK). The following day, the sections were incubated with a biotinylated secondary antibody (1:500, ZSGB-BIO, China) for 1 hour. Finally, DAB was used for color development (ZSGB-BIO, China), and counterstaining was performed with hematoxylin. Images were captured using a microscope (Olympus, Japan).

### Cell culture and model establishment

Primary microglia were isolated from 1-day-old (P1) SD rats and digested with collagenase and trypsin (Gibco, USA). Cells were cultured in DMEM medium (Gibco, USA) supplemented with 10% fetal bovine serum (FBS) (FBS, Gibco, USA) and 1% penicillin-streptomycin (Gibco, USA). To simulate the HIE condition, cells were exposed to an oxygen and glucose deprivation (OGD) environment, followed by reoxygenation treatment (36).

BV2 microglial cells were obtained from the Cell Bank of the Chinese Academy of Sciences. These cells were cultured in high-glucose DMEM (Gibco, USA) containing 10% FBS and 1% penicillin-streptomycin solution (Gibco, USA) under 37°C and 5% CO_2_ in a humidified incubator (Thermo Fisher Scientific, USA). When cells reached approximately 80-90% confluence, they were digested with 0.25% trypsin (Gibco, USA) for passage or further experimental procedures.

### Cell transfection

To overexpress RORα in BV2 microglia, cells were infected using a lentiviral vector. The lentiviral vector carrying the RORα overexpression fragment was purchased from GeneChem (China). Cells were cultured in 6-well plates until reaching 70-80% confluence, followed by infection at a multiplicity of infection (MOI) of 10. Polybrene (5 μg/mL, Sigma-Aldrich, USA) was added during infection to enhance transduction efficiency. After 48 hours, stable RORα-expressing cell lines were selected using puromycin (2 μg/mL, Sigma-Aldrich, USA). The expression of RORα in these cell lines was then verified by Western Blot (WB) analysis.

To knock down RORα expression, specific small interfering RNA (siRNA) was used for gene silencing. RORα-targeting siRNA (Santa Cruz Biotechnology, USA) and control siRNA (Santa Cruz Biotechnology, USA) were transfected using Lipofectamine RNAiMAX reagent (Invitrogen, USA). The sequence for RORα siRNA was as follows: sense (5’-3’): UCGCACCUGGAAACCUGCCAAUACUTT; antisense: AGUAUUGGCAGGUUUCCAGGUGCGATT. The control siRNA sequence was: sense (5’-3’): UUCUCCGAACGUGUCACGUTT; antisense: ACGUGACACGUUCGGAGAATT. Cells were seeded in 6-well plates, and once 70% confluence was achieved, 50nM of siRNA was mixed with Lipofectamine RNAiMAX and added to the cells. After 48 hours of transfection, the gene knockdown efficiency was assessed using Reverse Transcription Quantitative Polymerase Chain Reaction (RT-qPCR) and WB analysis.

### Cell counting kit-8 cell proliferation assay

To assess cell proliferation, we employed the CCK-8 (CCK-8, Dojindo, Japan). Cells from each group were seeded in 96-well plates at a density of 5 × 10³ cells per well. After 48 hours of incubation, 10μL of CCK-8 reagent was added to each well, and optical density (OD) was measured at a wavelength of 450nm using a microplate reader (BioTek, USA).

### Flow cytometry analysis of apoptosis

To assess the effects of various treatments on apoptosis, we used an Annexin V-FITC/PI Apoptosis Detection Kit (Beyotime, China). Following transfection or infection, cells were gently detached with trypsin, collected, and washed twice with PBS. The cells were then incubated with Annexin V-FITC and PI dyes for 15 minutes, following the manufacturer’s instructions. Apoptotic cells, including early and late apoptosis stages, were quantified using flow cytometry (BD Biosciences, USA), and data were analyzed with FlowJo software.

### Transmission electron microscopy analysis

To observe the occurrence of mitophagy, TEM was employed. Cells from each group were collected and fixed with 2.5% glutaraldehyde (Sigma-Aldrich, USA), followed by dehydration through an ethanol gradient. The samples were embedded in Epon 812 resin, and ultrathin sections (70 nm) were prepared. Mitochondrial morphology was then observed and imaged using a JEM-1400 Transmission Electron Microscope (JEOL, Japan).

### Mitophagy inhibition experiment

To investigate the role of mitophagy in the RORα-mediated signaling pathway, we used 3-MA (3-MA, Sigma-Aldrich, USA) as an autophagy inhibitor. After transfecting cells with the RORα overexpression plasmid, 3-MA (5mM) was added, and cells were incubated for 24 hours. Following this treatment, WB analysis was conducted to assess the expression levels of LC3-II, P62, and Beclin-1 proteins to evaluate the effect of autophagy inhibition. For *in vivo* validation, rats in the HIE + RORα + 3-MA group received an intraperitoneal injection of 3-MA (20 mg/kg) 30 minutes prior to HIE modeling (37).

### ROS detection (Dichlorofluorescein Diacetate staining)

ROS levels were assessed both *in vivo* and *in vitro* using DHE and DCFH-DA staining, respectively. Briefly, frozen brain tissue sections were incubated with 16 µM Dihydroethidium (DHE; 50102ES02, Yeasen, China) for 30 minutes at 37°C in the dark. For *in vitro* assays, cells were incubated with 10 μM DCF-DA (Beyotime, China) for 30 minutes after transfection or infection. After washing with PBS, fluorescence intensity was visualized using a Leica fluorescence microscope (Germany). ROS levels were semi-quantitatively analyzed using ImageJ software to compare differences across groups (38, 39).

### JC-1 Staining for mitochondrial membrane potential

To assess MMP, a JC-1 staining kit (Beyotime, China) was used. After treatment, cells were incubated with JC-1 staining solution for 20 minutes, followed by washing with PBS. The changes in the red-to-green fluorescence ratio were then observed under a fluorescence microscope (Leica Microsystems, Germany). Mitochondria with high membrane potential exhibited red fluorescence, while those with low membrane potential showed green fluorescence. Semi-quantitative analysis was performed using ImageJ software.

### RT-qPCR detection

To analyze the mRNA expression of RORα and related genes, total RNA was extracted from the brain tissues of HIBD patients and HIE rat models using Trizol reagent (Invitrogen, USA). RNA purity and concentration were measured with a Nanodrop 2000 spectrophotometer (Thermo Fisher Scientific, USA). The RNA was reverse-transcribed into cDNA using the PrimeScript RT Reagent Kit (Takara, Japan). RT-qPCR was performed with SYBR Green PCR Master Mix (Thermo Fisher Scientific, USA) on an Applied Biosystems QuantStudio 3 PCR system. Each sample was analyzed in triplicate, and expression levels were normalized to GAPDH using the 2^-ΔΔCt^ method. The primer sequences used were as follows: RORα (Forward: 5’-CACCAGCATCAGGCTTCTTTCC-3’, Reverse: 5’-GTATTGGCAGGTTTCCAGATGCG-3’) and GAPDH (Forward: 5’-GTCTCCTCTGACTTCAACAGCG-3’, Reverse: 5’-ACCACCCTGTTGCTGTAGCCAA-3’).

### WB analysis

WB was performed on cell and tissue samples from each group. Protein extraction was conducted using RIPA lysis buffer (Beyotime, China), and protein concentrations were quantified using the BCA assay (Thermo Fisher Scientific, USA). Proteins were then separated by SDS-PAGE on a 10% gel and transferred to a PVDF membrane (Millipore, USA). The membranes were incubated with primary antibodies against RORα (Abcam, UK, ab256799, 1:1000), cGAS (Abcam, UK, ab224144, 1:1000), STING (Cell Signaling Technology, USA, 13647, 1:1000), NLRP3 (Novus Biologicals, USA, NBP2-12446, 1:1000), Beclin-1 (Abcam, UK, ab302669, 1:1000), LC3-II (Abcam, UK, ab192890, 1:1000), P62 (Abcam, UK, ab109012, 1:1000), P21 (Abcam, UK, ab109199, 1:1000), P16 (Abcam, UK, ab51243, 1:1000), Caspase-1 (Abcam, UK, ab207802, 1:1000), GAPDH (Abcam, UK, ab8245, 1:1000). The secondary antibody, HRP-conjugated goat anti-rabbit IgG (Cell Signaling Technology, USA, 7074, 1:2000), was then applied. Protein bands were detected using an ECL reagent (Thermo Fisher Scientific, USA) and visualized with the Bio-Rad ChemiDoc MP imaging system (Bio-Rad, USA).

### Enzyme-linked immunosorbent assay

The levels of inflammatory cytokines IL-1β and Tumor Necrosis Factor-alpha (TNF-α) in each group were measured using ELISA kits (Thermo Fisher Scientific, USA). Following the manufacturer’s protocol, OD values were read at a wavelength of 450 nm. Each sample was tested in triplicate to ensure accuracy.

### Detection of mtDNA Release

Free mtDNA from rat brain tissue was extracted using the DNeasy Blood & Tissue Kit (QIAGEN, Germany). Approximately 20–30mg of brain tissue was processed according to the manufacturer’s instructions. Briefly, the tissue sample was homogenized in a lysis buffer and then digested with Proteinase K to release free mtDNA. Following digestion, the sample underwent a series of centrifugation and buffer exchange steps to isolate and purify the DNA. The extracted DNA was then eluted in 50μL of elution buffer. RT-qPCR was used to quantify the free mtDNA in brain tissues from different groups, with the following primer sequences for tRNA: (F: 5’-GGACATCATCGCCTGTGGCTTT-3’, R: 5’-AGTCGCTGTCAGTGAAGCCGAA-3’).

### Data analysis

All experiments were performed in triplicate, with data presented as mean ± standard deviation. Statistical analyses were conducted using GraphPad Prism 8 software (GraphPad Software, USA). Differences between groups were assessed using one-way analysis of variance (ANOVA), with a *p*-value < 0.05 considered statistically significant.

## Results

### Identification of key targets for HIE through transcriptomic screening

HIE poses a significant risk of mortality or severe disability. Globally, approximately 15 million people suffer from strokes each year, with around 33% resulting in death and another 33% leading to permanent disability. Despite extensive efforts to reduce stroke incidence and mortality, the overall number of cases continues to rise (40). Identifying new therapeutic targets and developing innovative treatments are crucial for improving HIE prevention and treatment.

**Figure 1:**
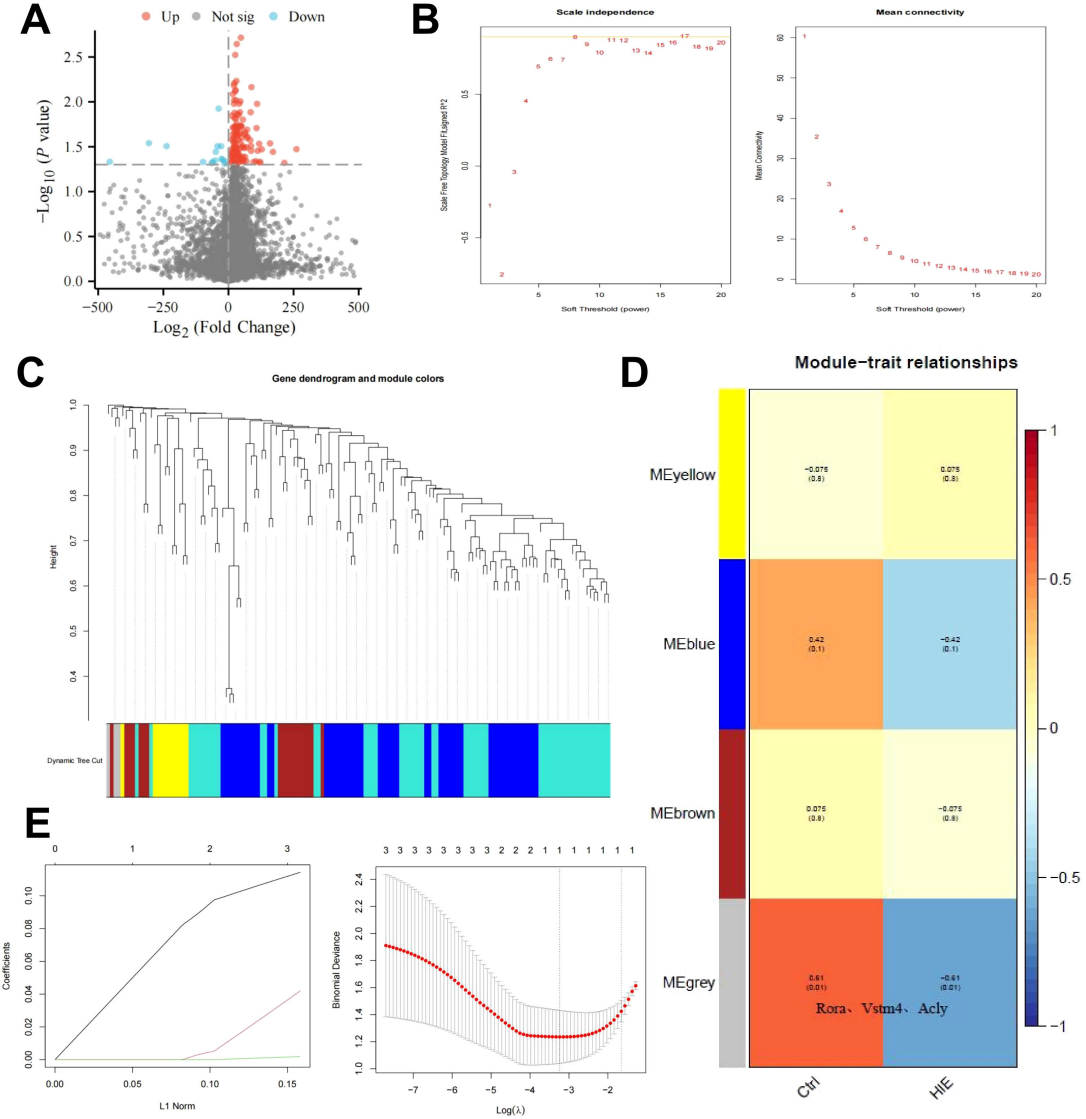
WGCNA and machine learning identify key targets affecting HIE. **(A)** Volcano plot of differential gene analysis between control and HIE brain tissues; **(B)** Scale independence, mean connectivity, and scale-free topology plot with weighted value β = 8, meeting scale-free network criteria; **(C)** Cluster dendrogram of co-expression network modules; **(D)** Correlation between gene modules obtained from clustering and HIE; **(E)** Distribution of LASSO coefficients for DEGs (left) and selection of the optimal parameter (lambda) for the LASSO model (right). The sample size for each group is n = 8.

To investigate the underlying mechanisms of HIE, we constructed an HIE rat model and used scRNA-seq to examine gene expression profiles. After normalizing the data, we performed differential expression analysis using the limma package in R, identifying 142 differentially expressed genes (DEGs), including 22 downregulated and 120 upregulated genes (**Figure 1A**). GO and KEGG pathway analyses revealed that these genes are primarily involved in immune activation, cellular response, metabolic regulation, and signaling pathways, notably inflammation-related pathways such as TNF and IL-17 (**Supplementary Figures S4A, B**). These findings highlight the pronounced activation of immune and inflammatory responses in the brains of HIE-affected rats, as well as alterations in energy metabolism and circadian regulation.

To further identify key therapeutic targets for HIE, we conducted a WGCNA based on differentially expressed mRNA data. Using the R software, we calculated the soft-threshold power (β) and determined that the optimal β value, satisfying the scale-free network criteria, was 8 (**Figure 1B**). Based on this soft threshold, we set the minimum module size to 9 and the module merging threshold to 0.2 to perform dynamic tree cutting (**Figure 1C**), which identified four gene modules: MEyellow, MEblue, MEbrown, and MEgrey. Notably, the MEgrey module generally represents genes that do not belong to any specific module.

Subsequently, we used these three clustered modules to explore their correlation with HIE as a clinical trait. The results revealed that MEgrey had the strongest correlation with HIE, with a correlation coefficient of -0.61 and a *p*-value below 0.01, and this module contains Rora, the key gene that we focus on in subsequent analyses(**Figure 1D**). This suggests that although the genes in this module do not exhibit strong associations with other modules, they may display significant relevance under specific conditions, potentially indicating their roles in related BP or pathological states.

To further refine our selection, we focused on the genes in the MEgrey module with the highest correlation. Using a regression analysis model, we fitted the module genes to eliminate similar characteristic genes, applying the glmnet function for LASSO regression. The data were modeled as a binary classification problem, with class labels extracted from sample names as the response variable using regular expressions. Model evaluation was conducted by plotting the model objects and performing cross-validation with cv.glmnet to determine the optimal lambda value (**Figure 1E**). This analysis identified a single key feature, RORα. RORα, a member of the nuclear receptor superfamily, primarily functions as a transcription factor that binds DNA to regulate gene expression, influencing a range of physiological processes such as circadian rhythm, immune response, inflammation regulation, and lipid metabolism. Aberrant expression of RORα has been associated with autoimmune diseases, neurodegenerative conditions, and metabolic disorders.

These findings indicate that RORα is a critical target in HIE.

### RORα is significantly upregulated in the HIE rat model

**Figure 2:**
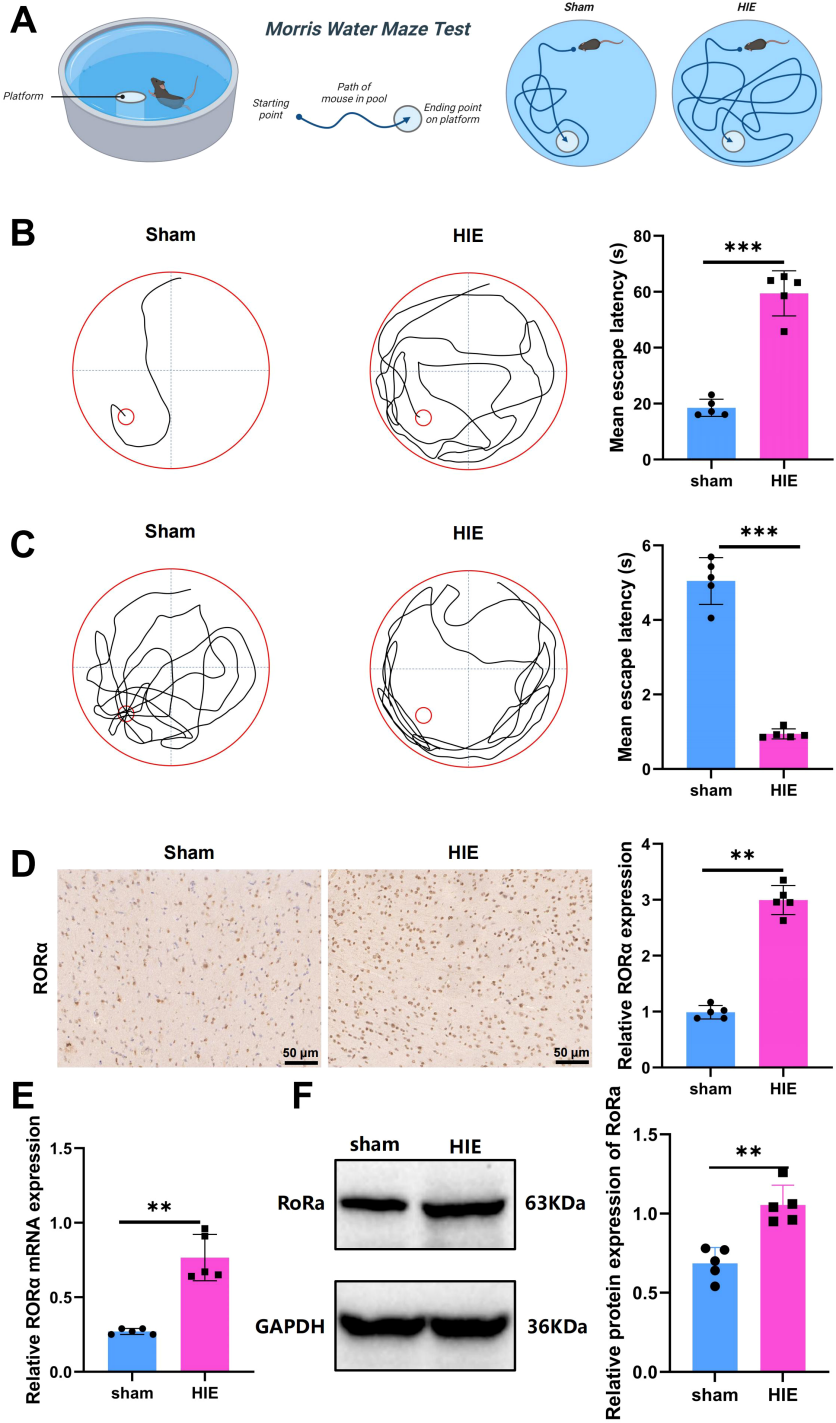
Significant upregulation of RORα in the HIE rat model. **(A)** Flowchart of the Morris water maze test; **(B)** Representative images of swimming paths and quantitative analysis of mean escape latency across groups; **(C)** Representative images of swimming paths after platform removal and quantitative analysis of the frequency of crossing the original platform location within 60 seconds; **(D)** IHC showing significant upregulation of RORα expression in the HIE rat model; **(E)** RT-qPCR analysis indicating a significant increase in RORα mRNA expression in the HIE rat model; **(F)** WB results showing upregulation of RORα protein expression, along with quantitative analysis. Data are presented as mean ± SEM, with N=5 animals per group. Statistical analysis was conducted using ANOVA followed by Tukey’s *post hoc* test. **p < 0.01, and ***p < 0.001.

A schematic of the Morris water maze test procedure is presented in **Figure 2A**. In the Morris water maze experiment, notable differences were observed between the Sham and HIE groups. As shown in **Figure 2B**, the Sham group demonstrated a significantly shorter latency to locate the platform compared to the HIE group. **Figure 2C** further illustrates that the HIE group exhibited poorer performance in locating the target platform area, with more random navigation patterns, while the Sham group followed a more direct path, indicating superior spatial memory and learning abilities. These findings suggest that the HIE model significantly impairs cognitive function in rats.

Using IHC, RT-qPCR, and WB analysis, we found that RORα was significantly upregulated in the HIE rat model. Immunohistochemical results revealed a 3.2-fold increase in RORα expression in the HIE model compared to the control group (**Figure 2D**).

RT-qPCR analysis further demonstrated a 2.8-fold increase in RORα mRNA levels in the HIE group (*p* < 0.001) (**Figure 2E**). Consistently, WB analysis indicated a 2.4-fold upregulation in RORα protein expression in the HIE model, aligning with the mRNA expression trend (**Figure 2F**).

These findings are consistent with transcriptome analysis results, further confirming that RORα is a key target in HIE.

### Single-cell analysis reveals the regulatory role of RORα in HIE

To further elucidate the regulatory role of RORα in HIE and to explore its regulatory pathways, we overexpressed RORα in HIE rat models and performed single-cell sequencing on brain tissue. After integrating the sample data using the Seurat package, we removed low-quality cells based on the criteria of 200 < nFeature_RNA < 5000 and percent.mt < 15%, resulting in an expression matrix of 19,498 genes and 37,278 cells. The correlation analysis of sequencing depth showed that after filtering, the correlation coefficient between nCount_RNA and percent.mt was r = -0.14, and between nCount_RNA and nFeature_RNA was r = 0.87 (**Supplementary Figure S5A**), indicating that the filtered cell data was of high quality and suitable for further analysis.

**Figure 3:**
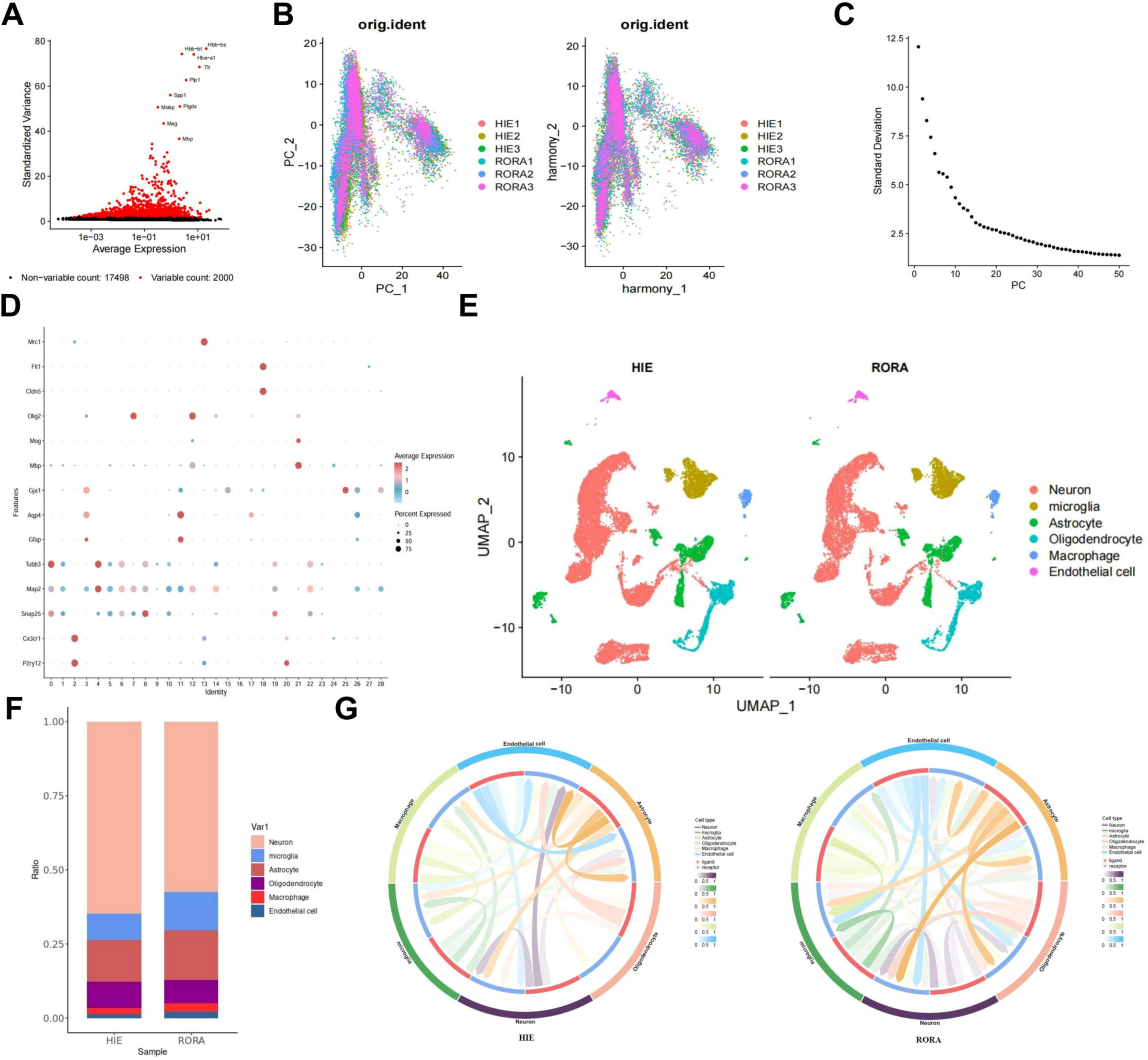
Single-cell analysis reveals the regulatory pathways of RORα in HIE. **(A)** Variance analysis identifies highly variable genes, with red indicating the top 2000 highly variable genes and black indicating low-variability genes; the top 10 highly variable genes are labeled. **(B)** Distribution of cells on PC_1 and PC_2 before and after batch correction, with each dot representing a cell. **(C)** Standard deviation distribution of PCs, where more important PCs show higher standard deviation. **(D)** Expression levels of known lineage-specific marker genes across different clusters, with red indicating high average expression and gray indicating low average expression. **(E)** Visualization of cell annotation results based on tSNE clustering, with each color representing a cell subgroup. **(F)** Bar chart showing the proportions of different cell types in HIE and RORα groups. **(G)** Cell-cell interaction networks across samples, with the outer ring colors indicating cell types, red in the inner ring representing cell receptors, blue indicating cell ligands, and line thickness reflecting interaction strength. n=3.

In the subsequent analysis of filtered cells, we screened for highly variable genes by gene expression variance, selecting the top 2,000 highly variable genes for downstream analysis (**Figure 3A**). Using the CellCycleScoring function, we calculated the cell cycle stages for each sample (**Supplementary Figure S5B**). After initial data normalization, we conducted PCA based on the selected highly variable genes to perform linear dimensionality reduction. The heatmap of key genes for PC_1 – PC_6 is shown (**Supplementary Figure S5C**), along with the distribution of cells across PC_1 and PC_2 (**Figure 3B**). Upon detecting batch effects, we applied the Harmony package for batch correction, which effectively minimized these effects for subsequent analyses (**Figure 3B**). Additionally, an ElbowPlot was used to rank PCs by standard deviation, demonstrating that PC_1 – PC_20 captured sufficient information to distinguish cells with significant analytical relevance (**Figure 3C**, **Supplementary Figure S5D**).

We applied the t-SNE algorithm to perform nonlinear dimensionality reduction on the top 30 PCs and selected a suitable resolution of 0.4 for cell clustering (**Supplementary Figure S5E**). This clustering analysis yielded 29 clusters, with gene expression profiles identified for each cluster (**Figure 3D**). After removing low-quality clusters (16, 24, and 27), we annotated the remaining clusters into six cell types—Neuron, Microglia, Astrocyte, Oligodendrocyte, Macrophage, and Endothelial cell—using the CellMarker online database (**Figure 3E**). Compared to the HIE group, we observed an increased proportion of Microglia in the RORα-overexpression group, which may suggest that RORα overexpression promotes the activation of these cells, potentially enhancing post-lesion clearance and repair mechanisms. Additionally, we found a relative reduction in the proportions of neurons and astrocytes, which may indicate that RORα overexpression reduces reactive astrocyte proliferation and scar formation, thereby potentially mitigating the pathological response triggered by HIE (**Figure 3F**).

We subsequently analyzed cell-cell interactions among these cells, revealing significant changes in the interaction intensity between microglia and other cell types in the RORα overexpression group, showing higher activity and a more extensive communication network compared to the HIE group. Additionally, astrocytes exhibited dense interactions in the HIE group, which were reduced in the RORα overexpression group, suggesting that RORα overexpression may modulate the activation state of these cells and their interactions with the surrounding environment (**Figure 3G**). These findings indicate that RORα overexpression may exert a notable influence on microglia, potentially enabling microglia-mediated interventions in HIE.

**Figure 4:**
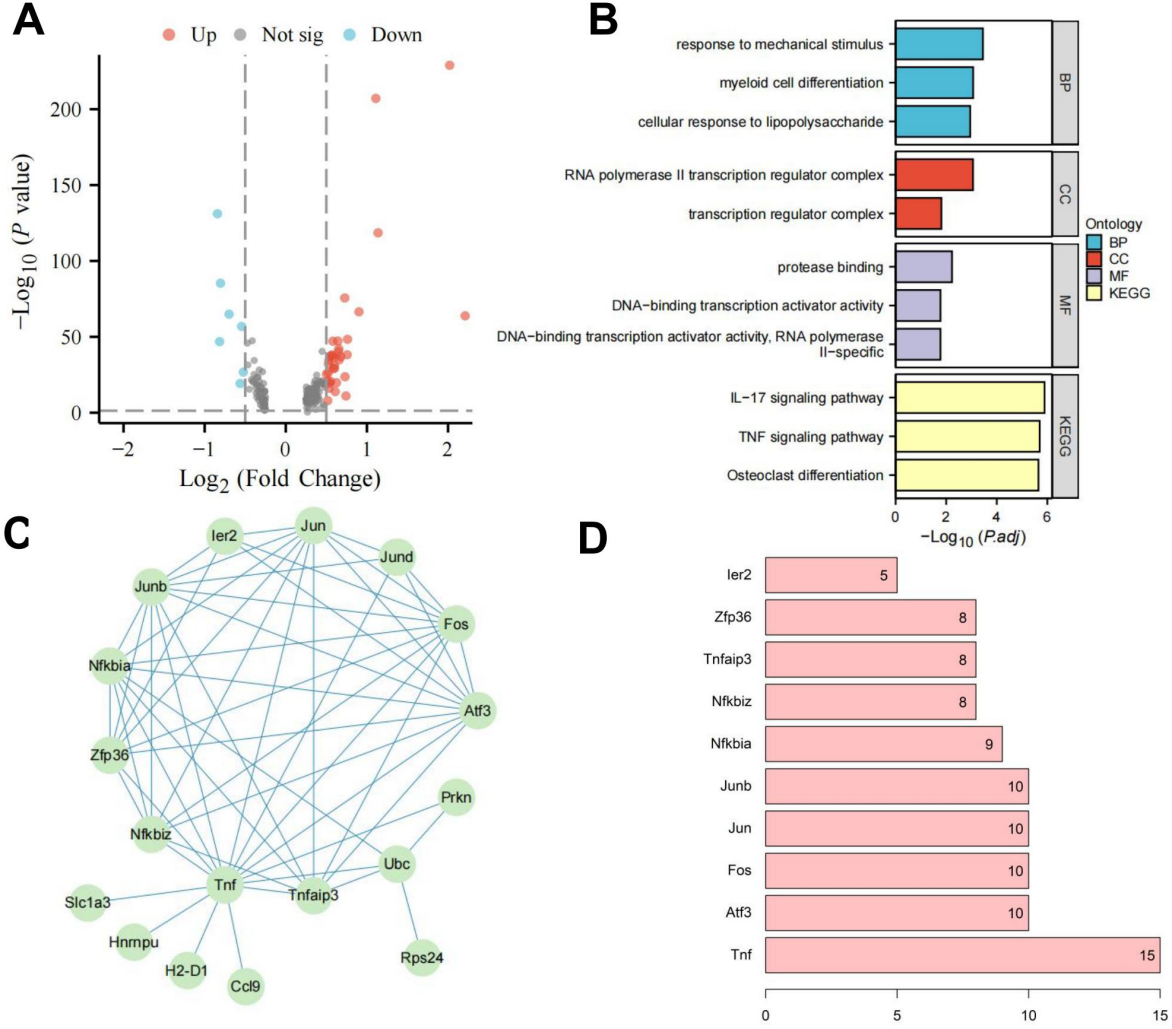
Investigation of RORα regulatory pathways in microglia. Note: **(A)** Volcano plot of DGE between the HIE and RORα groups; **(B)** Enrichment analysis of DEGs; **(C)** PPI network of DEGs; **(D)** Statistical analysis of PPI node connectivity, with the x-axis representing the number of connections (higher values indicate greater centrality). The sample size for each group: n=3.

To further investigate the regulatory pathways of RORα, we isolated microglia subpopulations and used the Seurat package’s FindMarkers function to analyze gene expression differences between the two groups, identifying 41 DEGs (**Figure 4A**). Enrichment analysis showed that these genes are closely associated with inflammatory responses, particularly through the regulation of the TNF and IL-17 signaling pathways (**Figure 4B**). We then used the STRING database to construct a PPI network for these 41 factors and assessed their centrality. The results indicated that the Tnf gene had the highest centrality (**Figures 4C, D**), highlighting Tnf as a key regulator of inflammatory responses and suggesting that RORα may influence HIE by modulating microglial inflammatory activity.

### RORα alleviates damage in an HIE cell model by suppressing inflammatory responses and activating mitophagy

**Figure 5:**
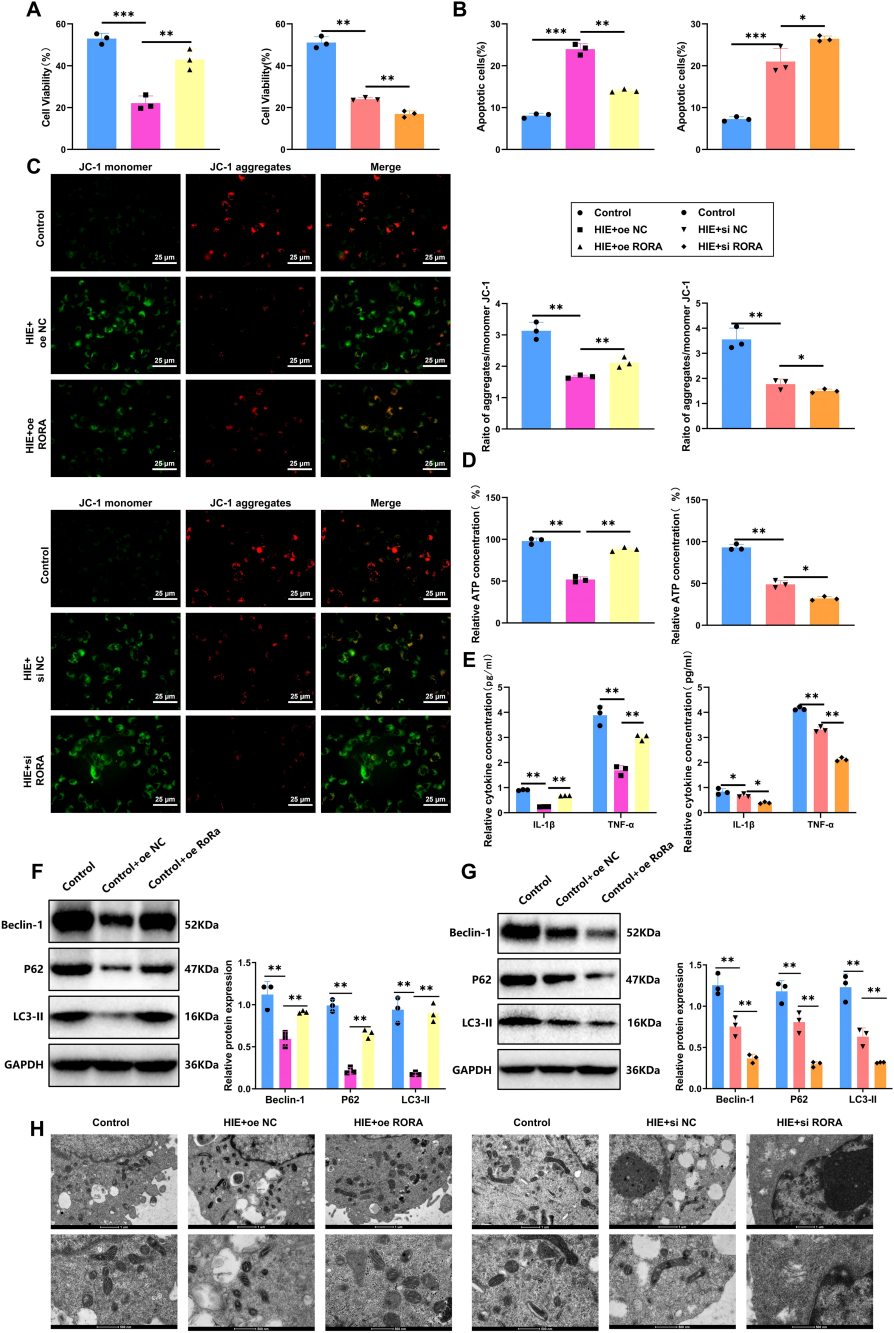
RORα alleviates damage in HIE cell models by suppressing inflammation and activating mitophagy. **(A)** Cell proliferation measured by CCK-8 assay across groups. **(B)** Apoptosis rates of each treatment group were assessed by flow cytometry. **(C)** MMP was evaluated by JC-1 staining across groups. **(D)** Mitochondrial ATP production in each group was measured by Seahorse XF24 analyzer. **(E)** Levels of inflammatory cytokines (IL-1β and TNF-α) in cell culture media determined by ELISA. **(F, G)** Expression levels of mitophagy-related proteins (LC3-II, P62, Beclin-1) in each treatment group detected by WB. **(H)** TEM images show mitophagosome formation and mitochondrial structure in each group. All data are presented as mean ± SEM, with experiments repeated three times. Statistical analysis was performed using ANOVA followed by Tukey’s *post-hoc* test, *p < 0.05, **p < 0.01, and ***p < 0.001.

We established both RORα overexpression and knockdown in cells using lentiviral transduction and specific siRNA transfection, respectively. We first tested time-course transfection efficiency and found that both overexpression and knockdown peaked at 48 hours post-transfection (**Supplementary Figures S6A, B**). Therefore, this time point was selected for all subsequent experiments. RT-qPCR and WB confirmed the mRNA and protein expression levels of RORα at 48 hours in each group, with knockdown and overexpression efficiencies presented in **Supplementary Figures S6C, D**. In the HIE cell model established using the OGD/R method, we first evaluated the impact of RORα on cell proliferation through a CCK-8 assay. The results indicated that OGD/R treatment significantly inhibited microglial proliferation, reducing the proliferation rate of the HIE group by 42% compared to the control. However, in the RORα overexpression group, cell proliferation improved markedly, increasing by approximately 29% relative to the HIE group and nearing control levels. Conversely, the RORα knockdown group showed further inhibition of proliferation, with a reduction of about 20% compared to the HIE group (**Figure 5A**). These findings suggest that RORα overexpression significantly restores proliferation in the HIE cell model, while its inhibition exacerbates proliferative deficits.

Regarding apoptosis, we analyzed the apoptosis rates in each group using flow cytometry. The HIE group exhibited a threefold increase in early and late apoptotic cells. Notably, the RORα overexpression group showed a 40% decrease in apoptosis rate compared to the HIE group, indicating a strong anti-apoptotic effect. In contrast, the RORα inhibition group demonstrated a substantial increase in apoptosis, with a 25% rise relative to the HIE group (**Figure 5B**). These results indicate that RORα overexpression effectively reduces apoptosis in the HIE cell model, whereas its suppression intensifies apoptotic activity.

To further investigate the protective role of RORα in an HIE cell model, we assessed mitochondrial function using JC-1 staining and a Seahorse XF24 Analyzer. JC-1 staining revealed that OGD/R treatment significantly decreased MMP in the HIE group, reducing the red/green fluorescence ratio by approximately 51%. In the RORα overexpression group, the MMP was markedly restored, with the red/green fluorescence ratio increasing by about 35% compared to the HIE group. Conversely, in the RORα inhibition group, the MMP declined further, showing a decrease of about 21% relative to the HIE group (**Figure 5C**).

The Seahorse analysis corroborated these findings, showing a significant increase in ATP production in the RORα overexpression group, approximately 36% higher than in the HIE group, while mitochondrial function in the RORα inhibition group was notably impaired (**Figure 5D**). These results suggest that RORα overexpression effectively improves mitochondrial function in the HIE model, while RORα inhibition exacerbates mitochondrial damage.

We also evaluated inflammatory factor secretion levels in the culture medium via ELISA. OGD/R treatment led to a significant increase in IL-1β and TNF-α secretion in the HIE group, with levels rising approximately 2.9-fold and 1.8-fold, respectively. However, in the RORα overexpression group, inflammatory factor secretion was significantly reduced, with IL-1β and TNF-α levels decreasing by approximately 42% and 35% relative to the HIE group. In contrast, the RORα inhibition group exhibited a more pronounced inflammatory response, with inflammatory factor levels increasing by an additional 30% compared to the HIE group (**Figure 5E**).

Additionally, the WB results (**Figure 5F**) indicate that overexpression of RORα significantly activates mitophagy, as evidenced by the increased protein levels of LC3-II, P62, and Beclin-1 compared to the HIE group. In contrast, the expression of these mitophagy-related proteins is markedly reduced in the RORα inhibition group.

TEM analysis further confirms that cells in the RORα overexpression group show an increase in mitophagosome formation and maintain a more intact mitochondrial structure. In contrast, mitochondria in the RORα inhibition group exhibit pronounced damage and a lack of autophagosomes (**Figures 5G, H**).

These findings suggest that RORα alleviates damage in the HIE cell model by activating mitophagy and reducing the inflammatory response.

### RORα inhibits NLRP3 inflammasome activation by suppressing the cGAS-STING signaling pathway through mitophagy activation

The abnormal activation of the cGAS-STING pathway can exacerbate central nervous system (CNS) damage by inducing various forms of programmed cell death, such as autophagy, necroptosis, and pyroptosis (20). In HIE models, the cGAS-STING pathway significantly amplifies the inflammatory response in microglia and astrocytes, leading to increased tissue damage and apoptosis (18).

**Figure 6:**
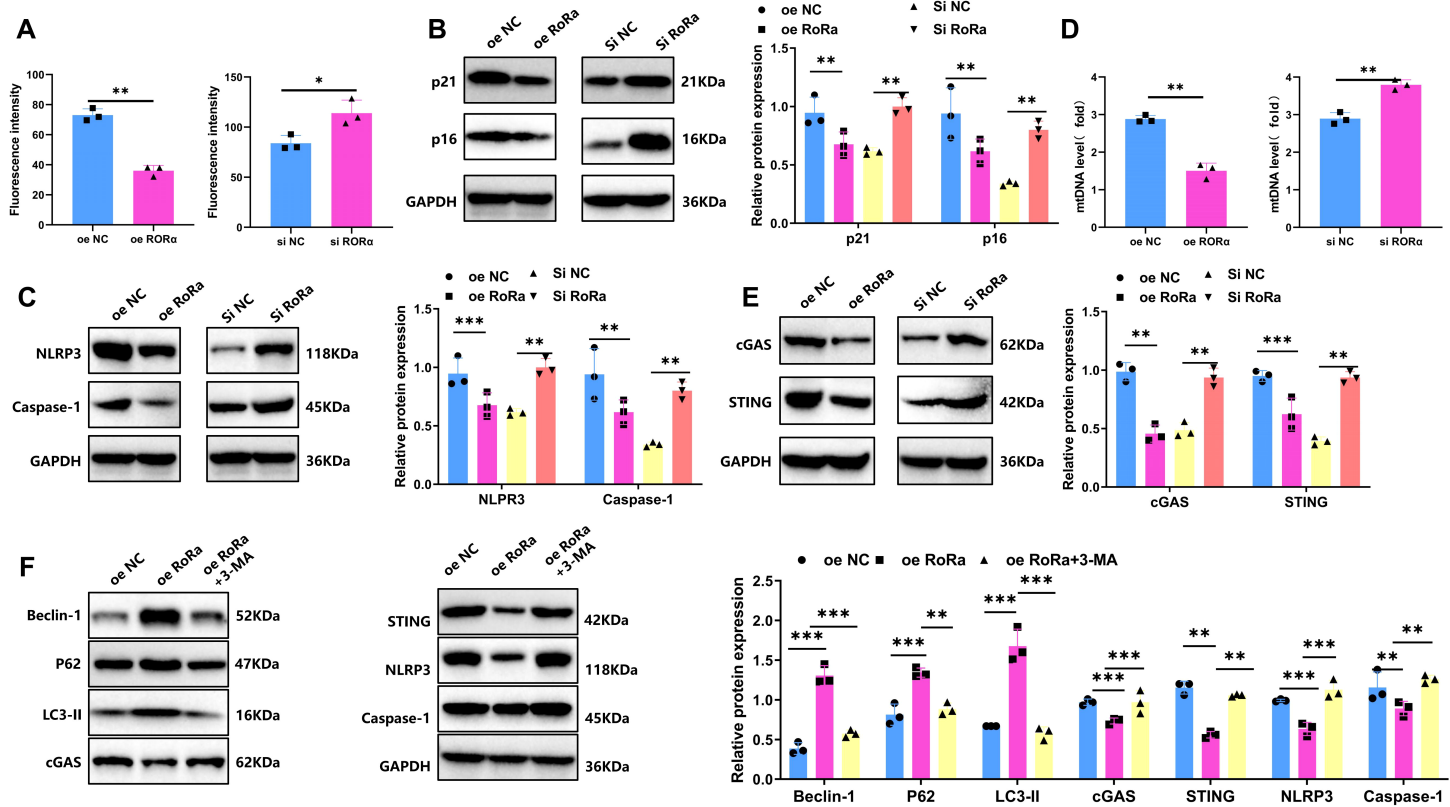
Overexpression of RORα suppresses ROS levels, reduces microglial senescence and inhibits NLRP3 Inflammasome Activation. **(A)** Intracellular ROS levels in the HIE group and treatment groups following OGD/R, detected by DCF-DA probe. **(B)** Expression levels of senescence markers p16 and p21 in the HIE and treatment groups after OGD/R, measured by WB. **(C)** Expression levels of NLRP3 inflammasome-related proteins NLRP3 and Caspase-1 in the HIE and treatment groups after OGD/R, analyzed by WB. **(D)** mtDNA release in the HIE and treatment groups following OGD/R, detected by RT-qPCR. **(E)** Expression levels of cGAS-STING pathway proteins (cGAS and STING) in the HIE and treatment groups after OGD/R, measured by WB. **(F)** Expression levels of LC3-II, P62, Beclin-1, and cGAS-STING-NLRP3-Caspase-1 proteins following treatment with 3-MA, analyzed by WB. Data are presented as mean ± SEM; experiments were repeated three times. Statistical analysis was conducted using ANOVA with Tukey’s *post hoc* test; *p < 0.05, **p < 0.01, ***p < 0.001.

In the HIE cell model, ROS levels in each group were measured using DCF-DA probes. Results indicated a significant increase in ROS levels in the HIE group, approximately 3.2-fold higher than in the control group (*p* < 0.001). In the RORα overexpression group, ROS levels were significantly reduced, decreasing by approximately 49% compared to the HIE group, while in the RORα knockdown group, ROS levels increased further by about 31% relative to the HIE group (*p* < 0.05) (**Figure 6A**). WB analysis showed that senescence markers p16 and p21 were markedly upregulated in the HIE group. In the RORα overexpression group, p16 and p21 levels decreased by approximately 40%, whereas in the RORα knockdown group, their expression increased by about 25% (**Figure 6B**).

Studies have reported that activation of the cGAS-STING pathway can induce NLRP3-mediated inflammation, contributing to neurotoxic responses (41). WB analysis of NLRP3 inflammasome-associated protein expression revealed significantly increased levels of NLRP3 and Caspase-1 in the HIE group. In the RORα overexpression group, the expression of these inflammasome-related proteins was significantly reduced, with NLRP3 and Caspase-1 levels decreasing by approximately 68% and 46%, respectively. Conversely, in the RORα knockdown group, the expression of NLRP3 and Caspase-1 further increased, with NLRP3 levels rising by approximately 31% (**Figure 6C**). These findings indicate that RORα activation can significantly suppress NLRP3 inflammasome activation, while RORα inhibition exacerbates inflammatory responses. These results highlight the critical role of RORα in reducing ROS levels, inhibiting senescence markers, and suppressing NLRP3 inflammasome activation, suggesting its potential as a neuroprotective target.

Previous studies have shown that mitochondrial dysfunction can lead to the release of mtDNA, which in turn activates the cGAS-STING signaling pathway (42). In the HIE group, increased mtDNA release was observed along with significantly upregulated expression of cGAS and STING. In contrast, the RORα overexpression group showed decreased mtDNA release and downregulation of cGAS and STING expression. On the other hand, the RORα knockdown group exhibited increased mtDNA release and further activation of the cGAS-STING signaling pathway (**Figure 6D, E**).

We next verified the role of mitophagy in RORα regulation of the cGAS-STING and NLRP3 inflammasome pathways using 3-MA. The experimental groups included oe NC, oe RORα, and oe RORα + 3-MA. As shown in **Figure 6F**, the oe RORα group exhibited significant mitophagy activation (evidenced by increased levels of LC3-II, P62, and Beclin-1), along with reduced cGAS and STING levels and inhibited activation of NLRP3 and Caspase-1. In the oe RORα + 3-MA group, mitophagy was notably suppressed (with decreased levels of LC3-II, P62, and Beclin-1), while levels of cGAS and STING markedly increased, along with elevated NLRP3 and Caspase-1 expression. These findings indicate that RORα overexpression suppresses the activation of the cGAS-STING and NLRP3 pathways by promoting mitophagy, while 3-MA inhibition of mitophagy significantly reverses this suppression.

Through this experiment, we preliminarily validated *in vitro* that RORα suppresses the NLRP3 inflammasome cascade by activating mitophagy and thereby inhibiting the cGAS-STING signaling pathway.

### RORα improves cognitive and motor functions in HIE rats through mitophagy activation

**Figure 7:**
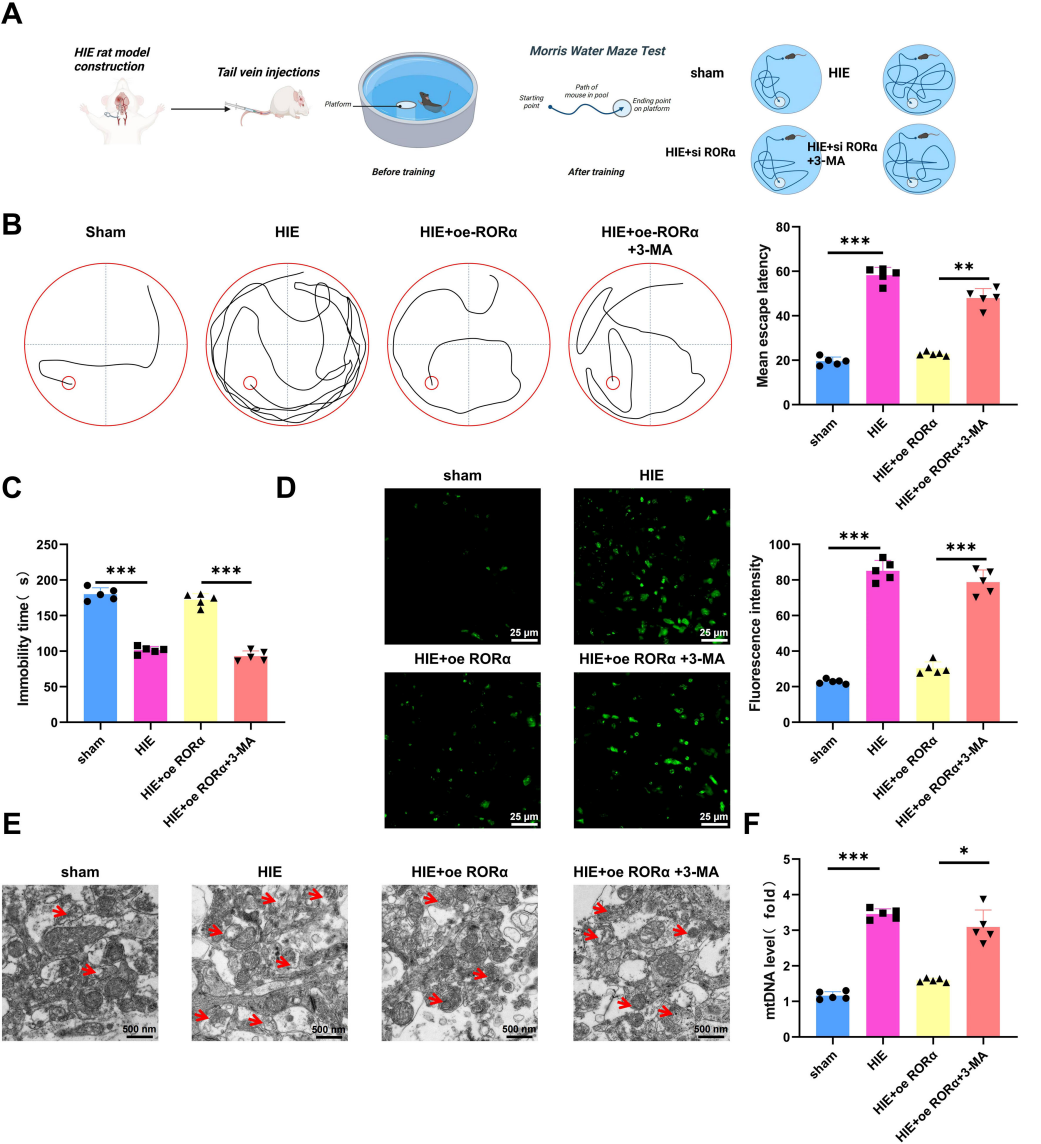
Effects of RORα overexpression and mitophagy inhibition on cognitive function, motor ability, ROS levels, and mitochondrial function in an HIE rat model. **(A)** Illustration of a Morris Water Maze Test. **(B)** Morris water maze test assessing cognitive function in HIE and treatment groups, measured by the average time to find the platform; **(C)** Tail suspension test evaluating motor ability in HIE and treatment groups, indicated by the total active time; **(D)** DHE staining assessing ROS levels in brain tissue from the HIE group and various treatment groups; **(E)** TEM imaging showing mitochondrial structural changes in brain tissue of HIE and treatment groups; **(F)** RT-qPCR analysis of free mtDNA release in brain tissue of HIE and treatment groups. Data are presented as mean ± SEM, with N=5 per group. Statistical analysis was performed using ANOVA followed by Tukey’s *post hoc* test, where *p < 0.05, **p < 0.01, and ***p < 0.001.

In this study, we evaluated the effects of RORα expression and Mitophagy inhibition on cognitive function, motor ability, ROS levels, and mitochondrial function in a HIE rat model, with an overview of the experimental procedure shown in **Figure 7A**.

The Morris water maze test indicated that rats in the HIE group exhibited severe cognitive impairment, with an average platform-finding time approximately twice as long as that of the control group, suggesting a substantial decline in spatial learning ability. However, overexpression of RORα significantly restored cognitive function, reducing the platform-finding time by approximately 40% compared to the HIE group, bringing it closer to control levels. Conversely, the use of 3-MA prolonged the platform-finding time again to near HIE group levels (**Figure 7B**), highlighting the critical role of Mitophagy in RORα-mediated cognitive recovery.

The tail suspension test further validated the changes in motor function. Rats in the HIE group showed a significant reduction in activity time—about 50% lower than the control group—indicating severe motor impairment. After RORα overexpression, motor function improved markedly, with activity time increasing by approximately 30% compared to the HIE group, reflecting partial restoration of motor ability. In the 3-MA group, motor function declined again, suggesting that the beneficial effects of RORα on motor function are dependent on Mitophagy (**Figure 7C**).

DHE staining revealed that ROS levels in the brain tissue of the HIE group, approximately 4.1 times higher than in the control group, indicating a key role for oxidative stress in the pathophysiology of HIE. Upon RORα overexpression, ROS levels significantly decreased, approximately 73% lower than in the HIE group and approaching control levels, suggesting that RORα effectively alleviates oxidative stress. Following the application of 3-MA, ROS levels increased again by approximately 53% compared to the RORα overexpression group (**Figure 7D**), underscoring the crucial role of mitophagy in controlling oxidative stress.

Further assessment of mitochondrial function supports these findings. TEM observations showed considerable mitochondrial damage in the brain tissue of the HIE group, characterized by mitochondrial swelling and cristae disruption. Activation of RORα markedly improved mitochondrial structure, reducing both swelling and cristae damage, indicating restoration of mitochondrial function. However, in the 3-MA group, mitochondrial damage worsened, with more pronounced swelling and cristae disruption (**Figure 7E**), highlighting the importance of mitophagy in maintaining mitochondrial integrity and function.

RT-qPCR analysis of mtDNA release revealed a significant increase in free mtDNA in the brain tissue of the HIE group, approximately 2.5 times higher than in the control group, indicating that mitochondrial damage is associated with extensive mtDNA release. RORα overexpression significantly reduced mtDNA release by about 43% compared to the HIE group, nearing control levels, further confirming mitochondrial function restoration. In the 3-MA group, mtDNA release increased again, approximately 1.1 times higher than in the RORα overexpression group (**Figure 7F**), underscoring the critical role of mitophagy in regulating mtDNA release.

## Discussion

This study is the first to reveal the anti-inflammatory mechanism of RORα in HIE, opening new avenues for HIE treatment. HIE is a severe neurological disorder that often leads to long-term neurodevelopmental impairment in newborns, significantly affecting their quality of life. Previous research has primarily focused on the roles of apoptosis and oxidative stress pathways in HIE, with limited exploration of the mechanisms involving microglia (9, 43). Additionally, although mitophagy has been shown to play a crucial regulatory role in various inflammatory diseases, systematic studies on its role in HIE are lacking (12, 44). This study innovatively employs multi-omics analysis to reveal the mechanism of RORα in microglia, demonstrating that it alleviates inflammation by activating mitophagy and suppressing ROS production. This finding not only enriches the understanding of HIE’s molecular mechanisms but also provides a new therapeutic target for intervention. By integrating multi-omics approaches—including scRNA-seq, WGCNA analysis, and the LASSO regression model—this research enhances scientific rigor and systematic insight, identifying RORα as a key therapeutic target.

In this study, RORα was identified as a critical target for HIE, with its central role in regulating microglial inflammation demonstrated through multi-omics analysis. Previous studies have confirmed the anti-inflammatory and metabolic regulatory functions of RORα in other neurodegenerative diseases, but its role in HIE remains largely unexplored (30, 45–47). Using WGCNA, we identified the MEgrey module as closely related to HIE and ultimately pinpointed RORα as a potential target through the LASSO regression model. Compared to traditional target screening methods, multi-omics analysis enhances accuracy in gene selection by effectively eliminating irrelevant genes, enabling more precise identification of key targets. Our findings suggest that RORα may exert anti-inflammatory effects in microglia by modulating the cGAS-STING pathway and NLRP3 inflammasome cascade, offering a novel molecular perspective and potential intervention target for HIE research.

This study found that RORα significantly suppresses ROS production and NLRP3 inflammasome activation by activating mitophagy in microglia. Mitophagy, a cellular defense mechanism that clears damaged mitochondria and reduces ROS production, has been noted in other neurological disorders; however, its specific role in HIE remains unclear (25, 26, 48, 49). This study is the first to confirm that RORα-activated mitophagy decreases the release of mtDNA from damaged mitochondria, thereby inhibiting activation of the cGAS-STING pathway in microglia. This mechanism offers a novel perspective on inflammation regulation in HIE, validating the anti-inflammatory role of mitophagy in this context. Furthermore, RORα regulates inflammatory responses at multiple levels in microglia through mitophagy, demonstrating a significant anti-inflammatory effect in the HIE model. Unlike conventional methods that target ROS reduction alone, RORα achieves multifaceted inflammation control through mitophagy.

The cGAS-STING signaling pathway, a key inflammatory pathway, plays a critical role in innate immunity, yet its specific mechanisms in HIE remain underexplored. This study is the first to link the anti-inflammatory role of RORα with the cGAS-STING pathway, revealing an innovative mechanism in which activation of mitophagy reduces mtDNA release, thereby inhibiting this pathway. Aberrant activation of the cGAS-STING pathway can trigger various inflammatory responses, and in HIE, this activation may exacerbate inflammatory damage in microglia. Our findings indicate that RORα reduces the activity of the cGAS-STING pathway through mitophagy activation, thereby lowering the activation of the NLRP3 inflammasome. This discovery not only deepens the understanding of the cGAS-STING pathway in HIE-related inflammation but also proposes a potential strategy for HIE intervention by targeting this pathway, offering new molecular insights for HIE treatment.

In both *in vivo* and *in vitro* experiments, RORα overexpression demonstrated significant anti-inflammatory effects in HIE models. Using the Morris water maze and tail suspension tests, we evaluated the impact of RORα overexpression on cognition and motor function in HIE rats. The results showed substantial improvement, underscoring RORα’s neuroprotective potential. Additionally, *in vitro* experiments confirmed the role of RORα overexpression in suppressing inflammatory cytokine expression and reducing ROS production, further supporting its therapeutic potential in HIE. Compared to conventional single-drug interventions, RORα overexpression exhibits a more comprehensive anti-inflammatory effect by simultaneously inhibiting ROS and suppressing inflammasome cascades. These findings suggest that RORα offers broad neuroprotective benefits, presenting significant potential for future applications in HIE therapy.

This study further confirmed the dependency of RORα’s anti-inflammatory effects on mitophagy using 3-MA. The experimental results showed that the use of 3-MA partially reversed RORα’s anti-inflammatory effects, indicating that RORα’s anti-inflammatory mechanism largely relies on the activation of mitophagy. This finding provides key evidence for understanding the mechanistic role of RORα in HIE, suggesting that mitophagy not only plays a crucial role in regulating ROS production and inflammatory factors but also may inhibit the cGAS-STING pathway and NLRP3 inflammasome activation. Compared to strategies that solely inhibit ROS or inflammatory factors, activating mitophagy for comprehensive anti-inflammatory effects offers a more systematic regulatory approach for treating HIE and other inflammatory neuropathies.

The role of RORα in regulating inflammation in HIE revealed in this study highlights its high potential for clinical translation. Current treatments for HIE are limited to symptomatic relief and are unable to effectively halt disease progression, while RORα regulation provides a new molecular target for HIE therapy. Through *in vivo* and *in vitro* experiments, we demonstrated RORα’s regulatory effects on ROS and NLRP3 inflammasome in microglia, offering theoretical support for future clinical applications. As an endogenous regulatory factor, RORα modulates multi-level inflammatory responses through mitophagy activation, potentially offering greater safety and efficacy compared to exogenous drugs. Consequently, RORα holds promise as an effective intervention for HIE, providing a novel approach to improving patient outcomes.

Despite the promising protective effects of RORα in HIE pathology revealed in this study, several challenges remain for clinical translation. For instance, efficiently and safely activating RORα without causing adverse effects in other neurological or immune functions requires further exploration. Moreover, the complex regulatory roles of RORα in various cell types may involve intricate interactions. Future studies should examine the dose-response effects, safety, and long-term impact of RORα, optimize drug delivery systems through preclinical trials, and investigate potential combination therapies to advance the clinical application of RORα in HIE and other central nervous system disorders.

## Conclusion

This study, using multi-omics analysis, reveals the critical role of RORα in HIE. We found that RORα significantly improves microglial function in HIE by activating mitophagy, which reduces the release of mtDNA from damaged mitochondria. This reduction, in turn, decreases the activation of the cGAS-STING pathway and suppresses the overactivation of the NLRP3 inflammasome, thereby alleviating the excessive inflammatory response associated with HIE pathology (Graphic abstract). Additionally, the results show that RORα enhances microglial proliferation by promoting mitophagy, reducing oxidative stress damage in cells, and providing essential protection for neuronal survival and brain repair. These findings offer new evidence for the therapeutic potential of RORα in HIE, suggesting that RORα may be a key regulatory factor in brain injury and neuroprotection.

Our study unveils the multi-layered protective mechanisms of RORα in neonatal HIE, particularly its role in modulating the cGAS-STING and NLRP3 cascades via mitophagy, identifying a novel molecular target. Given the limited treatment options for central nervous system diseases like HIE, the therapeutic strategy proposed here to activate RORα provides a promising approach for controlling neuroinflammation and potentially improving long-term outcomes in HIE patients. Clinically, this discovery could drive the development of novel RORα agonists or combination therapies to regulate mitophagy and mitigate post-injury neuroinflammation.

## Data Availability

The original contributions presented in the study are publicly available. This data can be found here: NCBI BioProject PRJNA1227688.

## Supplementary material

Supplementary Figure 1 Schematic diagram of HIE rat model establishment.

Supplementary Figure 2 Flowchart of gene expression study in the brain tissue of HIE rats based on scRNA-seq and bioinformatics analysis.

Supplementary Figure 3 Workflow for scRNA-seq and bioinformatics analysis in HIE Rats.

Supplementary Figure 4 GO and KEGG enrichment results of DEGs. Note: **(A)** GO enrichment results of DEGs; **(B)** KEGG enrichment results of DEGs. The sample size for each group is n = 8.

Supplementary Figure 5 Re-clustering analysis of cell subpopulations based On Pca dimensionality reduction, batch correction, and cell-type identification. Note: **(A)** Scatter plots showing correlations between nCount_RNA and percent.mt, and between nCount_RNA and nFeature_RNA after filtering; **(B)** Cell cycle phase distribution; **(C)** Heatmaps of the top 20 genes contributing to PCs 1–6, with yellow indicating upregulation and purple indicating downregulation; **(D)** Batch correction using Harmony, where the x-axis indicates interaction iterations; **(E)** Clustering of cells under different resolution settings. Sample size: n = 3 per group.

Supplementary Figure 6 Verification of RORα overexpression and knockdown. Note: **(A)** Time-course analysis of RORα mRNA expression in oe NC and oe RORα groups following lentiviral transduction; **(B)** in si NC and si RORα groups following siRNA transfection; **(C)** Changes in relative RORα protein levels among the control group (NC), RORα overexpression group, and RORα knockdown group; **(D)** Changes in relative RORα mRNA levels among the control group (NC), RORα overexpression group, and RORα knockdown group. All data are presented as mean ± standard error, with experiments repeated three times. Statistical analysis was performed using ANOVA followed by Tukey’s *post hoc* test, **p* < 0.05, ***p* < 0.01, and ****p* < 0.001.

## Glossary

ANOVA: Analysis of Variance
BP: Biological Processes
CC: Cellular Components
cGAS: Cyclic GMP-AMP Synthase
CCK-8: Cell Counting Kit-8
DCF-DA: Dichlorofluorescein Diacetate
DEG: Differentially Expressed Gene
DGE: Differential Gene Expression
ELISA: Enzyme-Linked Immunosorbent Assay
FBS: Fetal Bovine Serum
GO: Gene Ontology
HIE: Hypoxic-Ischemic Encephalopathy
IHC: Immunohistochemistry
KEGG: Kyoto Encyclopedia of Genes and Genomes
LASSO: Least Absolute Shrinkage and Selection Operator Regression
MAD: Median Absolute Deviation
MF: Molecular Functions
MOI: Multiplicity of Infection
MMP: Mitochondrial Membrane Potential
mtDNA: Mitochondrial DNA
MMP: Mitochondrial Membrane Potential
OGD: Oxygen and Glucose Deprivation
OD: Optical Density
PCA: Principal Component Analysis
PCs: Principal Components
PPI: Protein-Protein Interaction Networks
RIN: RNA Integrity Number
rRNA: Ribosomal RNA
RORα: Retinoic Acid Receptor-Related Orphan Receptor Alpha
ROS: Reactive Oxygen Species
RT-qPCR: Reverse Transcription Quantitative Polymerase Chain Reaction
scRNA-seq: Single-Cell RNA Sequencing
SD: Sprague-Dawley
siRNA: Small Interfering RNA
STING: Stimulator of Interferon Genes
TEM: Transmission Electron Microscopy
TNF-α: Tumor Necrosis Factor-Alpha
WGCNA: Weighted Gene Co-Expression Network Analysis
WB: Western Blot 3-MA, 3-Methyladenine
