# Supplementary figures and images for: RORα-activated mitophagy attenuating hypoxic-ischemic encephalopathy via suppression of microglial cGAS-STING axis
Source: Front Immunol. 2025 Jul 29;16:1592737. doi: 10.3389/fimmu.2025.1592737 (PMC12341001; doi:10.3389/fimmu.2025.1592737)

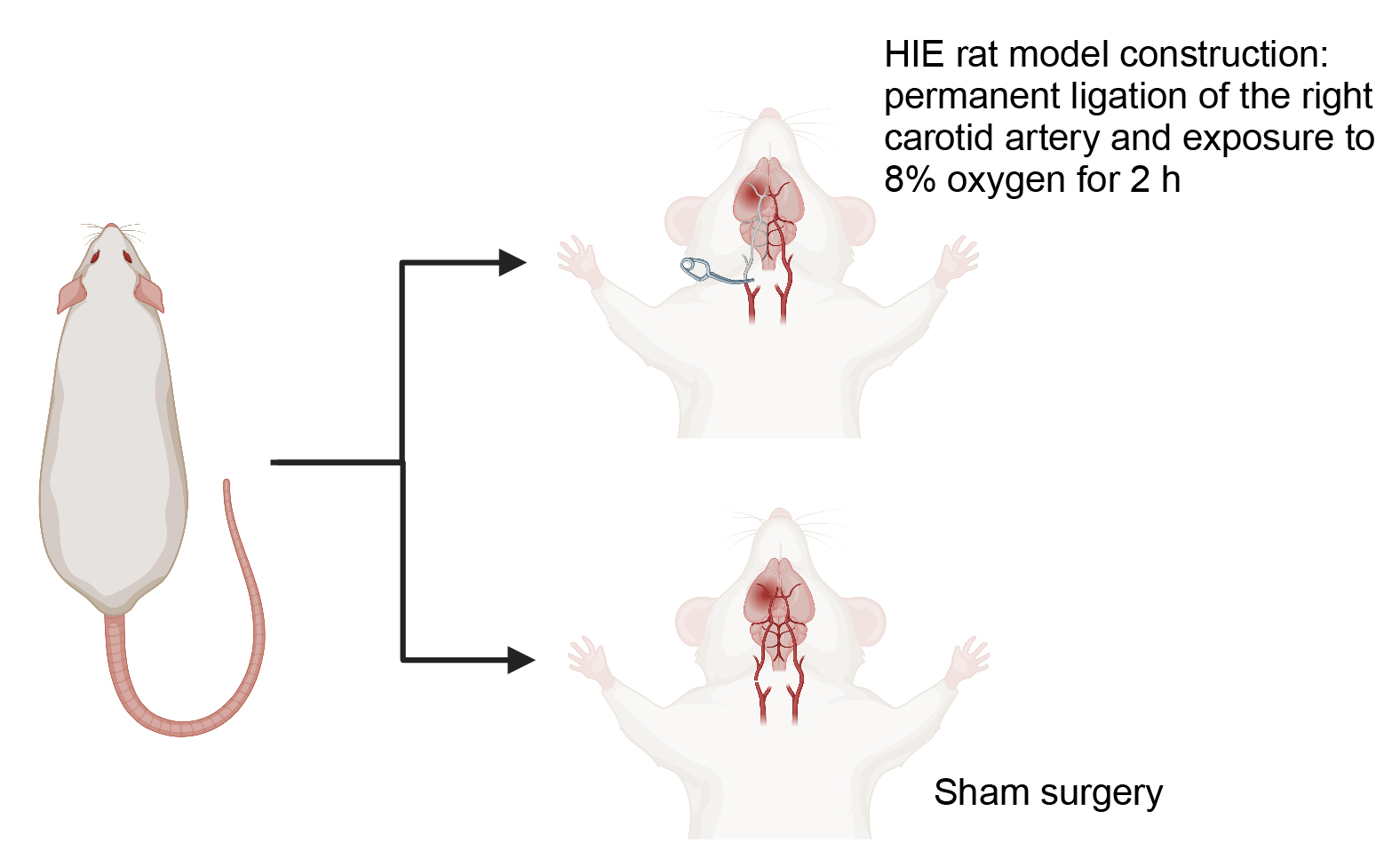

Supplement: Supplementary Figure 1 — Schematic diagram of HIE rat model establishment. [file Image1.jpeg]

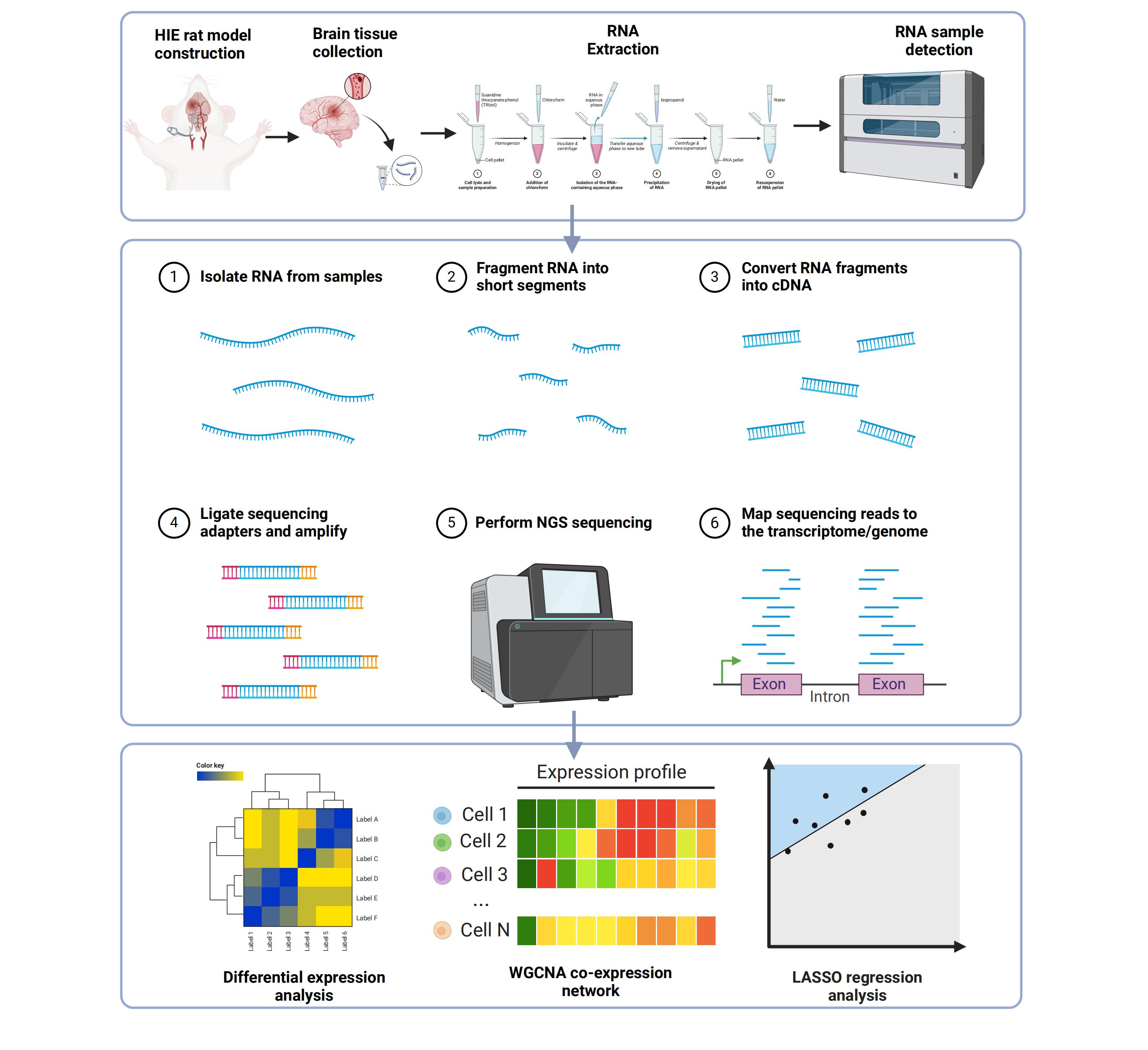

Supplement: Supplementary Figure 2 — Flowchart of gene expression study in the brain tissue of HIE rats based on scRNA-seq and bioinformatics analysis. [file Image2.jpeg]

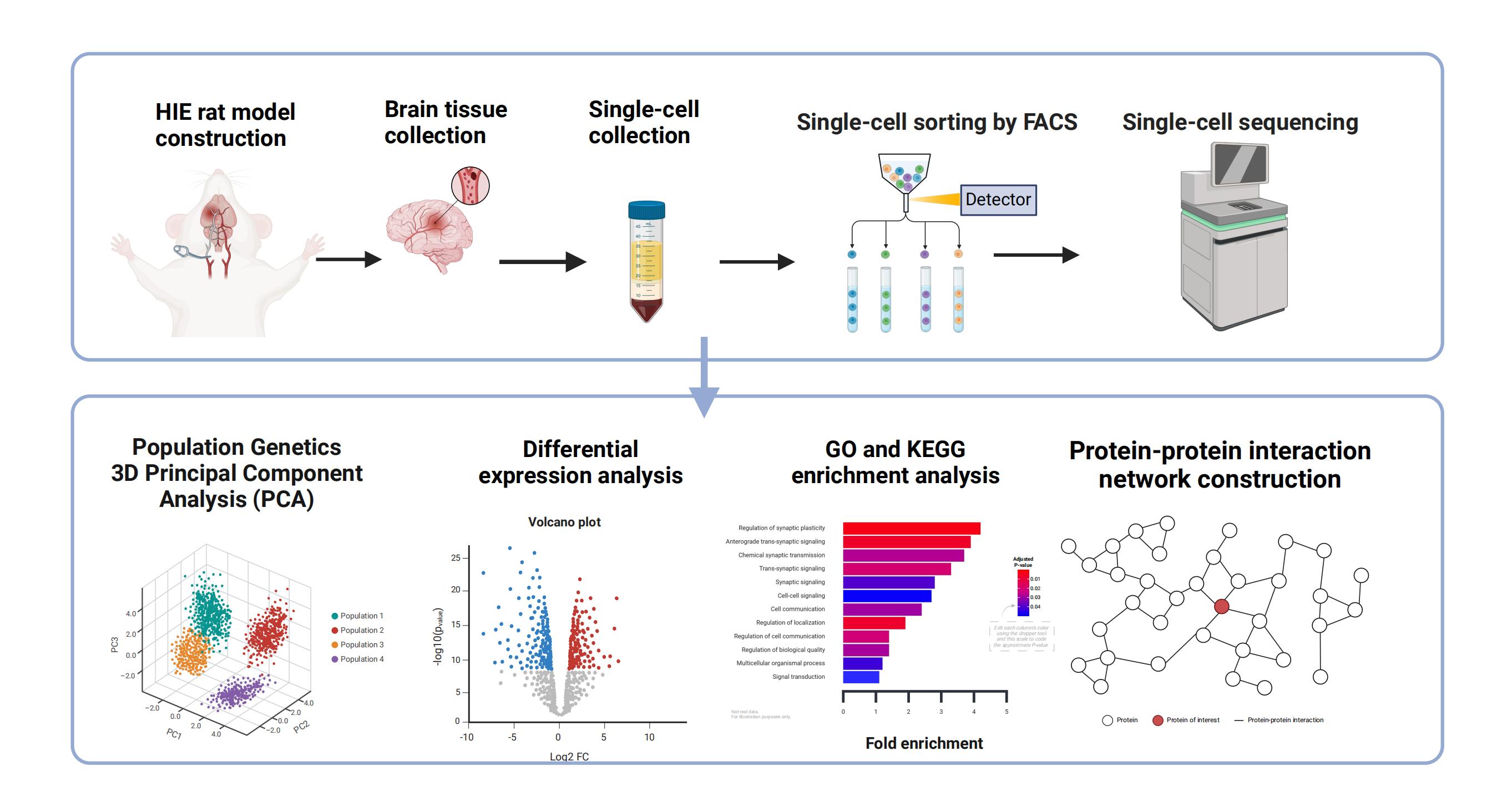

Supplement: Supplementary Figure 3 — Workflow for scRNA-seq and bioinformatics analysis in HIE Rats. [file Image3.jpeg]

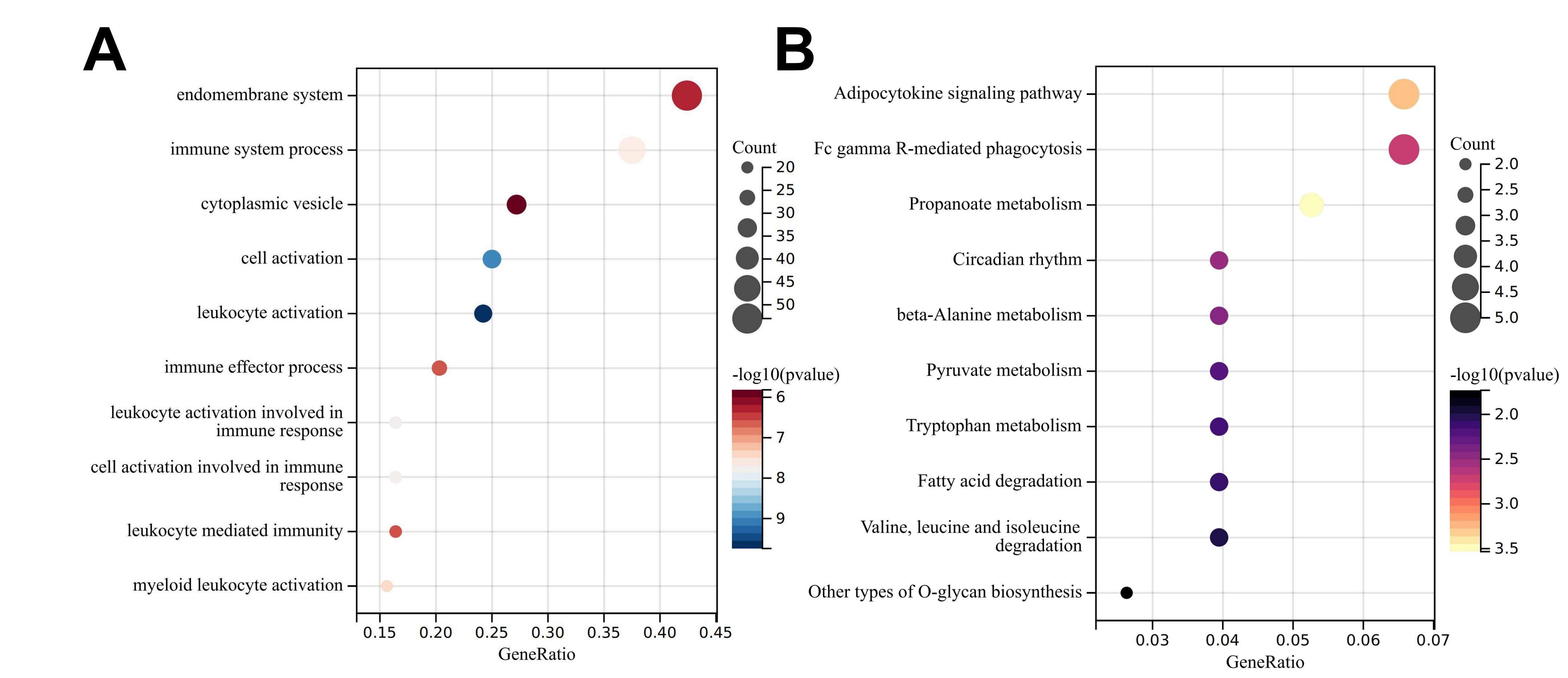

Supplement: Supplementary Figure 4 — GO and KEGG enrichment results of DEGs. Note: (A) GO enrichment results of DEGs; (B) KEGG enrichment results of DEGs. The sample size for each group is n = 8. [file Image4.jpeg]

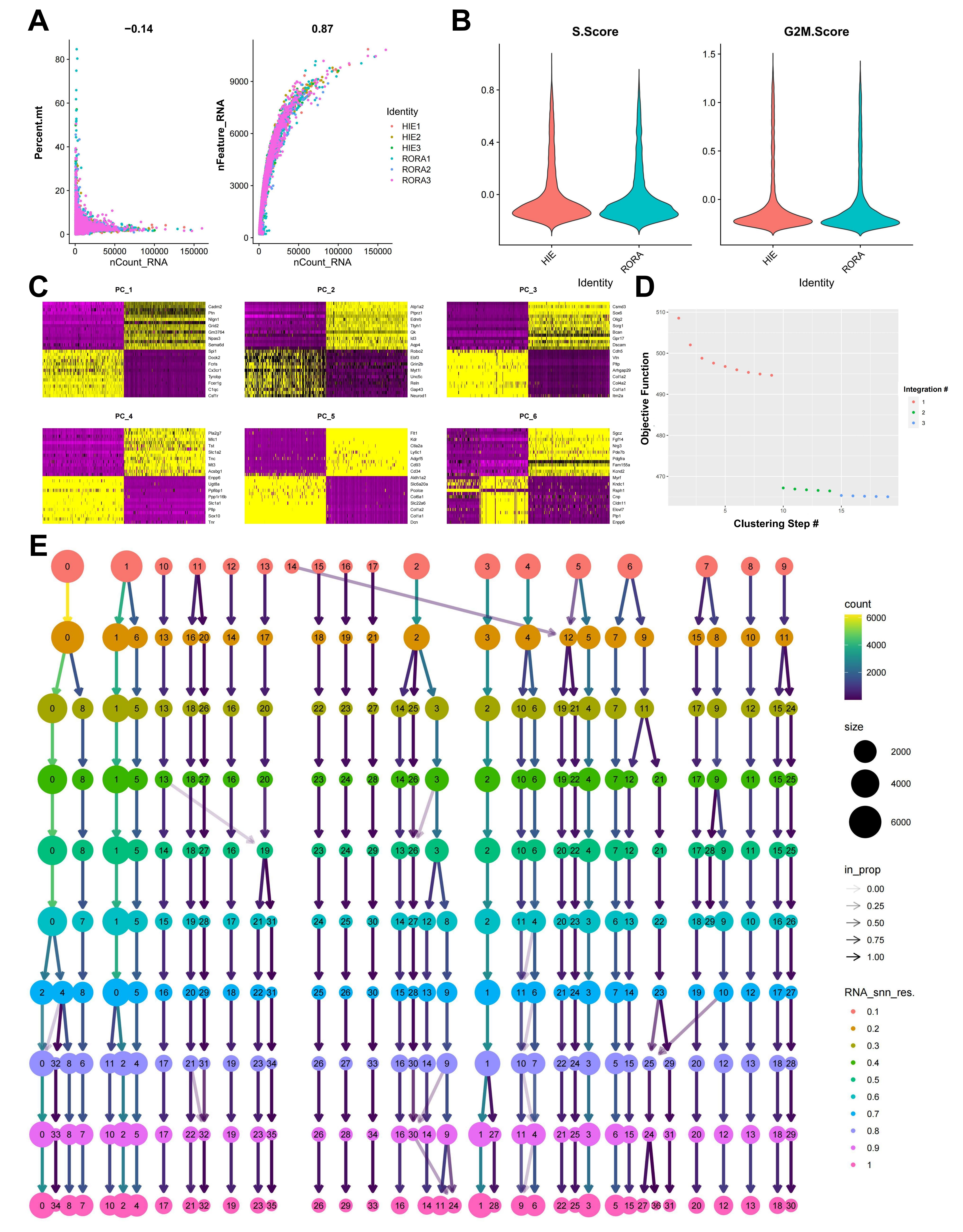

Supplement: Supplementary Figure 5 — Re-clustering analysis of cell subpopulations based On Pca dimensionality reduction, batch correction, and cell-type identification. Note: (A) Scatter plots showing correlations between nCount_RNA and percent.mt, and between nCount_RNA and nFeature_RNA after filtering; (B) Cell cycle phase distribution; (C) Heatmaps of the top 20 genes contributing to PCs 1–6, with yellow indicating upregulation and purple indicating downregulation; (D) Batch correction using Harmony, where the x-axis indicates interaction iterations; (E) Clustering of cells under different resolution settings. Sample size: n = 3 per group. [file Image5.jpeg]

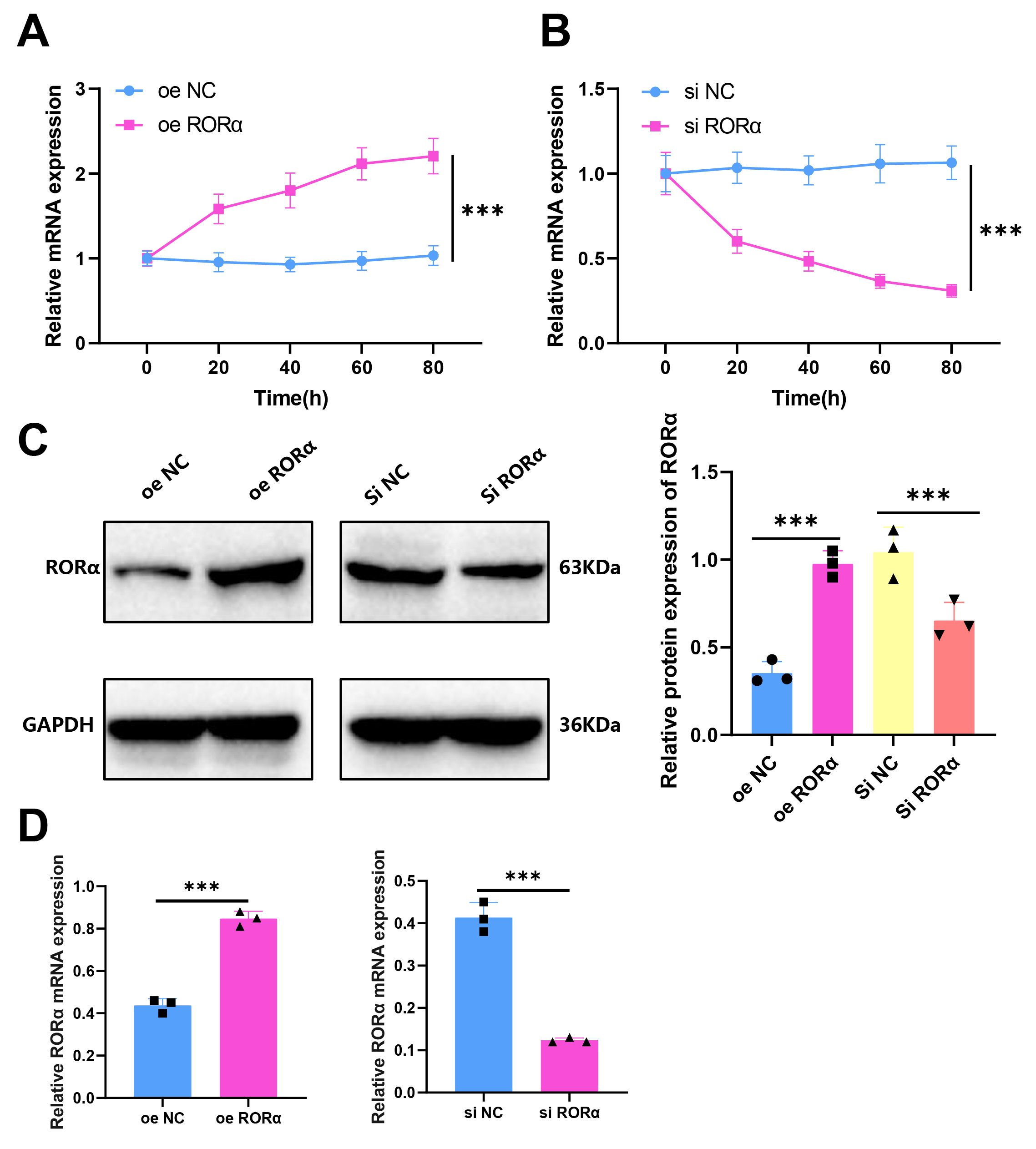

Supplement: Supplementary Figure 6 — Verification of RORα overexpression and knockdown. Note: (A) Time-course analysis of RORα mRNA expression in oe NC and oe RORα groups following lentiviral transduction; (B) in si NC and si RORα groups following siRNA transfection; (C) Changes in relative RORα protein levels among the control group (NC), RORα overexpression group, and RORα knockdown group; (D) Changes in relative RORα mRNA levels among the control group (NC), RORα overexpression group, and RORα knockdown group. All data are presented as mean ± standard error, with experiments repeated three times. Statistical analysis was performed using ANOVA followed by Tukey’s post hoc test, *p < 0.05, **p < 0.01, and ***p < 0.001. [file Image6.jpeg]
